# Differential Management of the Replication Terminus Regions of the Two *Vibrio cholerae* Chromosomes during Cell Division

**DOI:** 10.1371/journal.pgen.1004557

**Published:** 2014-09-25

**Authors:** Gaëlle Demarre, Elisa Galli, Leila Muresan, Evelyne Paly, Ariane David, Christophe Possoz, François-Xavier Barre

**Affiliations:** 1 CNRS, Centre de Génétique Moléculaire, Gif-sur-Yvette, France; 2 Université Paris-Sud, Orsay, France; A*STAR, Singapore

## Abstract

The replication terminus region (Ter) of the unique chromosome of most bacteria locates at mid-cell at the time of cell division. In several species, this localization participates in the necessary coordination between chromosome segregation and cell division, notably for the selection of the division site, the licensing of the division machinery assembly and the correct alignment of chromosome dimer resolution sites. The genome of *Vibrio cholerae*, the agent of the deadly human disease cholera, is divided into two chromosomes, chrI and chrII. Previous fluorescent microscopy observations suggested that although the Ter regions of chrI and chrII replicate at the same time, chrII sister termini separated before cell division whereas chrI sister termini were maintained together at mid-cell, which raised questions on the management of the two chromosomes during cell division. Here, we simultaneously visualized the location of the dimer resolution locus of each of the two chromosomes. Our results confirm the late and early separation of chrI and chrII Ter sisters, respectively. They further suggest that the MatP/*matS* macrodomain organization system specifically delays chrI Ter sister separation. However, TerI loci remain in the vicinity of the cell centre in the absence of MatP and a genetic assay specifically designed to monitor the relative frequency of sister chromatid contacts during constriction suggest that they keep colliding together until the very end of cell division. In contrast, we found that even though it is not able to impede the separation of chrII Ter sisters before septation, the MatP/*matS* macrodomain organization system restricts their movement within the cell and permits their frequent interaction during septum constriction.

## Author Summary

The genome of *Vibrio cholerae* is divided into two circular chromosomes, chrI and chrII. ChrII is derived from a horizontally acquired mega-plasmid, which raised questions on the necessary coordination of the processes that ensure its segregation with the cell division cycle. Here, we show that the MatP/*matS* macrodomain organization system impedes the separation of sister copies of the terminus region of chrI before the initiation of septum constriction. In its absence, however, chrI sister termini remain sufficiently close to mid-cell to be processed by the FtsK cell division translocase. In contrast, we show that MatP cannot impede the separation of chrII sister termini before constriction. However, it restricts their movements within the cell, which allows for their processing by FtsK at the time of cell division. These results suggest that multiple redundant factors, including MatP in the *enterobacteriaceae* and the Vibrios, ensure that sister copies of the terminus region of bacterial chromosomes remain sufficiently close to mid-cell to be processed by FtsK.

## Introduction

Most bacteria harbour a single chromosome and, in the rare case in which the genetic material is divided on several chromosomes, the extra-numerous ones appear to have derived from horizontally acquired mega-plasmids that subsequently gained essential genes [1]. This is notably the case for *Vibrio cholerae*, the agent of the deadly human diarrheal disease cholera, whose genome is divided between a 2.961 Mbp ancestral chromosome, chrI, and a 1.072 Mbp plasmid-derived chromosome, chrII [2]. The preferential transcription of chrII genes during colon colonization compared to *in vitro* growth under aerobic conditions suggests that this genomic organization is important for rapid adaptation to different environments [3]. Likewise, other bacteria harbouring multipartite genomes can adopt several different life cycles [4], [5], [6], [7]: the *rhyzobium*, the *burkholderia* and the *vibrio*, can alternatively spread freely in the environment or interact as symbionts or pathogens with eukaryotic cells; the *borrelia* are obligate parasites that need to infect several different eukaryotic organisms in the course of their life cycle. Thus, multipartite genomes seem to offer a selective advantage for the adaptation to very different environmental conditions. However, the necessary coordination between replication, chromosome segregation and cell scission raises questions on the management of the different chromosomes of such bacteria.

Bacterial chromosomes harbour a single origin of bidirectional replication and are generally circular. Replication ends in a region opposite of the origin of replication, the terminus region, in which is usually found a specific recombination site dedicated to the resolution of chromosome dimers, *dif* [8]. Fluorescent microscopic observation of chromosome segregation in mono-chromosomal bacteria revealed that it is concurrent with replication and starts with the active positioning of sister copies of the origin region into opposite cell halves [9], [10], [11]. As replication progresses along the left and right chromosomal arms, newly replicated loci are progressively segregated towards their future daughter cell positions. However, the mean time during which sister loci remain together before separation is variable [12]. In particular, sister copies of the terminus region co-localize at mid-cell until the initiation of cell division in *E. coli* and *P. aeruginosa* [9], [10], [13], [14]. This mode of segregation can participate in the coordination between chromosome segregation and cell division. Indeed, nucleoid occlusion factors impede the assembly of the cell division machinery until a time when the only genomic DNA left at mid-cell consist of the sister copies of the terminus region in *Escherichia coli* and *Bacillus subtilis* [15], [16]; the long co-localization of sister termini at mid-cell is at least in part dictated by the MatP/*matS* macrodomain organisation system in *E. coli* [17], [18]; a DNA translocase, FtsK, which is recruited to mid-cell as part of the divisome and which pumps chromosomal DNA in the orientation dictated by repeated polar motifs that point towards *dif*, the KOPS, promotes the orderly segregation of the DNA within the terminus region of *E. coli* chromosome [13], [19], [20]. One of the functions of FtsK is to control the resolution of chromosome dimers, which result from homologous recombination events between circular sister chromatids, by the addition of a cross-over between sister *dif* sites at the time of constriction [21]. FtsK is also thought to participate in sister chromatid decatenation [22], [23] and to create a checkpoint to delay constriction until sister terminus regions have been fully segregated [19], [24], [25].

**Figure 1:**
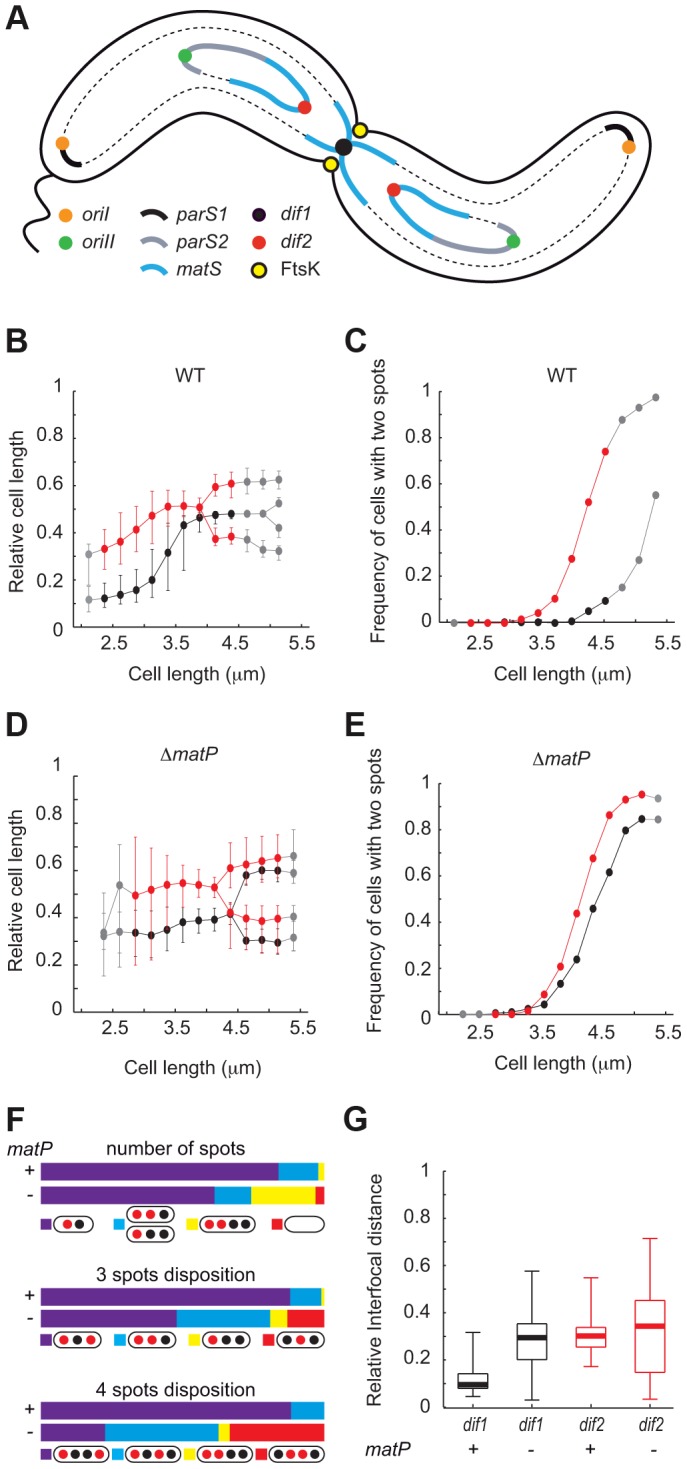
Early segregation of *V. cholerae* TerII. **A.** Schematic representation of the two sister chromatids of each of the two *V. cholerae* chromosomes during septation. **B.** and **D.** Relative position of *dif1* (in black) and *dif2* (in red) along the long axis of the cell as a function of cell length in WT (B) or Δ*matP* (D) background. **C.** and **E.** Frequency of cells with separated *dif1* (in black) and *dif2* (in red) sisters as a function of cell length in WT (C) or Δ*matP* (E) background. The plain red and black lines show the data for the bins containing at least 30 cells (see Figure S1); the dashed grey lines show the data for bins containing 3 to 29 cells (see Figure S1). **F.** Upper panel, relative number of cells with a single *dif1* and *dif2* spot (in purple), a single *dif1* spot and two *dif2* spots or a single *dif2* spot and two *dif1* spots (in blue) or two *dif1* spots and two *dif2* spots (in yellow). Cells without either of these numbers of spots were plotted in the ‘other’ category (in red). Middle panel, in the category of cells with 3 spots, relative number of cells with the depicted dispositions of spots in WT or Δ*matP* background. Lower panel, in the category of cells with 4 spots, relative number of cells with the depicted dispositions of spots in WT or Δ*matP* background. **G.** Interfocal distance of the sister copies of the *dif* locus of each of the two *V. cholerae* chromosomes, (*dif1* in black and *dif2* in red), as a function of cell length, in WT or Δ*matP* background.

*V. cholerae* chrI and chrII are circular and harbour a single *dif* site in the region opposite of their origin of replication, *dif1* and *dif2*, respectively (Figure 1A, [26]). Segregation of the two chromosomes is concurrent with replication and both chromosomes adopt a longitudinal organization within the cell [27]. However, chrII is replicated late in the C period of the cell cycle, when most of chrI has been replicated, and the initiation of its segregation is consequently delayed [28]. In addition, the origin region of chrI, OriI, locates to the old pole of newborn cells and one OriI sister migrates to the other pole after replication (Figure 1A, [27]). The origin region of chrII, OriII, locates to mid-cell in newborn cells and the two OriII sisters migrate towards the ¼ and ¾ positions after replication (Figure 1A, [27]). This is at least in part dictated by the presence of a partition machinery of a chromosomal type on chrI, *parABS1*, and a partition system that groups with plasmid and phage machineries on chrII, *parABS2* (Figure 1A, [27], [29], [30]). The last chromosomal regions to be segregated are the terminus regions of chrI and chrII, TerI and TerII, respectively (Figure 1A, [27]). Both TerI and TerII locate at or close to the new pole in newborn cells (Figure 1A, [27]). Replication termination of the two *V. cholerae* chromosomes is synchronous [28] and unreplicated TerI and TerII are recruited to mid-cell at approximately the same time (Figure 1A, [27]). *V. cholerae* is closely related to *E. coli* in the phylogenetic tree of bacteria and its genome harbour the same *dam* co-occurring DNA maintenance machineries as *E. coli* [31]. This includes a unique *E. coli* MatP ortholog and the presence of cognate *matS* sites in both TerI and TerII. In addition, a common pair of tyrosine recombinases, XerC and XerD, serves to resolve dimers of each of the two *V. cholerae* chromosomes despite the sequence divergence of *dif1* and *dif2* [26]. Dimer resolution is controlled by a unique *E. coli* FtsK ortholog, whose translocation activity is oriented by KOPS motifs that point towards the dimer resolution site of each of the two chromosomes [26]. By analogy to *E. coli*, MatP is thought to maintain sister copies of TerI and TerII at mid-cell and FtsK to promote the orderly segregation of the DNA within TerI and TerII. Correspondingly, the separation of sister copies of a locus situated at 40 kbp from *dif1* seemed coordinated with cell division (Figure 1A, [27], [32]). However, sister copies of a locus situated at 49 kbp from *dif2* separated before cell division, which questioned the role of FtsK and MatP on TerII segregation (Figure 1A, [32]).

The aim of this work was to identify the contribution of MatP to the segregation dynamics of TerI and TerII. We show by replication profiling that *dif1* and *dif2* are located next to the replication terminus of chrI and chrII, respectively. Simultaneous visualization of the positions of *dif1* and *dif2* within the cell then allowed us to confirm the late and early separation of TerI and TerII, respectively. However, we show that TerII sisters keep colliding with each other at mid-cell during constriction by genetically probing the relative frequency of sister chromatid contacts occurring at mid-cell at the time of cell division along the two chromosomes and by time-lapse fluorescent microscopy. We further show that the frequency of these collisions depends on the MatP/*matS* macrodomain organization system, possibly because it restricts the movements of TerII within the cell. We also show that MatP promotes the late mid-cell co-localization of TerI sisters. However, TerI loci remain in the vicinity of the cell centre and sister chromatid contacts remain frequent in its absence.

## Results

### Early TerII and delayed TerI segregation

Replication profiling of *V. cholerae* cells by deep sequencing indicated that termination most frequently occurred at a distance of ∼90 kbp and ∼70 kbp from the reference loci that had been used by Srivastava *et al.* for the simultaneous visualization of the positions of TerI and TerII (Figure S1A, [32]). It was therefore possible that the behaviour of these loci did not fully reflect TerI and TerII segregation dynamics. To confirm the segregation pattern of the terminus regions of chrI and chrII, we simultaneously visualized the intracellular location of *dif1* and *dif2* in cells that were exponentially growing in minimal media. We used the *lacO*/LacI-mCherry system to label the *dif1* locus and the pMT1 *parS*/yGFP-ParB system to label the *dif2* locus. Cells were classified according to their length in bins of 0.25 µm. They had a median length of 3.2 µm (Figure S2A). The smallest cells, i.e. the youngest cells, contained a single *dif1* spot at one of the two cell poles (Figure 1B). This pole, which results from the previous division event, is hereafter referred to as the new pole. The preferential localization of *dif1* towards the new pole was used to orientate the cells. A single *dif2* spot was also observed in the youngest cells (Figure 1B). This spot was located in the younger cell half, at an intermediate position between the *dif1* spot and the middle of the cell (Figure 1B). The polarity of the *dif1* and *dif2* spots decreased as a function of cell elongation and the median position of each spot reached mid-cell in cells of an intermediate length (Figure 1B). The majority of the longest cells, i.e. the closest to cell division, displayed a single *dif1* spot, which was located at mid-cell and was flanked by two *dif2* spots (Figure 1B). Indeed, <15% of the cells from the 4.25 µm–4.5 µm bin displayed two *dif1* spots whereas >80% of them displayed two *dif2* spots (Figure 1C). In addition, the proportion of cells containing two *dif2* spots reached 100% in the cells that were longer than 4.5 µm whereas only 50% of these cells displayed two *dif1* spots (Figure 1C, grey points). Marker frequency analysis indicated that the earlier timing of appearance of cells with two *dif2* foci was not due to an earlier timing of replication of *dif2* compared to *dif1* (Figure S1A). The same pattern of segregation was observed when the *dif1* and *dif2* labelling systems were switched, excluding any possible artefact linked to the visualization strategy (Figure S3). Finally, *dif2* sisters were found to segregate further away from each other and from mid-cell than *dif1* sisters (Figure 1G). Taken together, these results suggest that in the vast majority of cases TerII sisters separated before cell division whereas TerI sister separation was delayed until the end of cell division.

### MatP impedes *dif1* sister separation and constrains *dif2* positioning

We next investigated the influence of the MatP/*matS* macrodomain organization system on TerI and TerII segregation. *V. cholerae* cells in which MatP was disrupted were slightly longer than wild-type cells. In minimal medium, they had a median length of 3.77 µm (Figure S2B). Nevertheless, growth competition indicated that they lost less than 0.23% of fitness per generation (Figure S4). The smallest cells had a single *dif1* and a single *dif2* spot, which were both positioned closer to mid-cell than in wild-type cells (Figure 1D). This was accompanied by an increase in position variability (Figure 1D). As a consequence, mid-cell recruitment was no longer directly observable in cells of intermediate lengths (Figure 1D). In addition, the timing of separation of *dif1* spots was now very similar to the timing of separation of *dif2* spots (Figure 1E). Marker frequency analysis indicated that this was not due to a change in the relative replication timing of *dif1* and *dif2* (Figure S1B). Many cells of intermediate length now displayed two *dif1* and two *dif2* spots and most of the cells of the following bins had two *dif1* and two *dif2* spots (Figure 1E). This was directly reflected in the proportion of cells displaying a single *dif1* spot and a single *dif2* spot and the proportion of cells with two *dif1* and two *dif2* spots in the entire population (Figure 1F, number of spots). The separation of *dif2* sisters remained slightly ahead of the separation of *dif1* sisters (Figure 1E), which was reflected in the higher proportion of cells harbouring a single *dif1* spot and two *dif2* spots than cells harbouring a single *dif2* spot and two *dif1* spots (Figure 1F, 3 spots disposition). However, the disposition of spots became more random and many cells now displayed *dif2* spots more centrally located than *dif1* spots (Figure 1F, 3 spots disposition and 4 spots disposition). Finally, sister *dif* sites migrated to opposite cell halves after their separation (Figure 1D) and the distances between the sisters of both sites were similar (Figure 1G). Taken together, these results suggested that MatP contributed to the precise positioning of TerI before and after replication and that it delayed the separation of TerI sisters to the time of cell division. MatP also contributed to the precise positioning of TerII. However, it was unable to impede TerII sisters from separating before septum constriction.

### Monitoring TerI and TerII management during septum constriction

**Figure 2:**
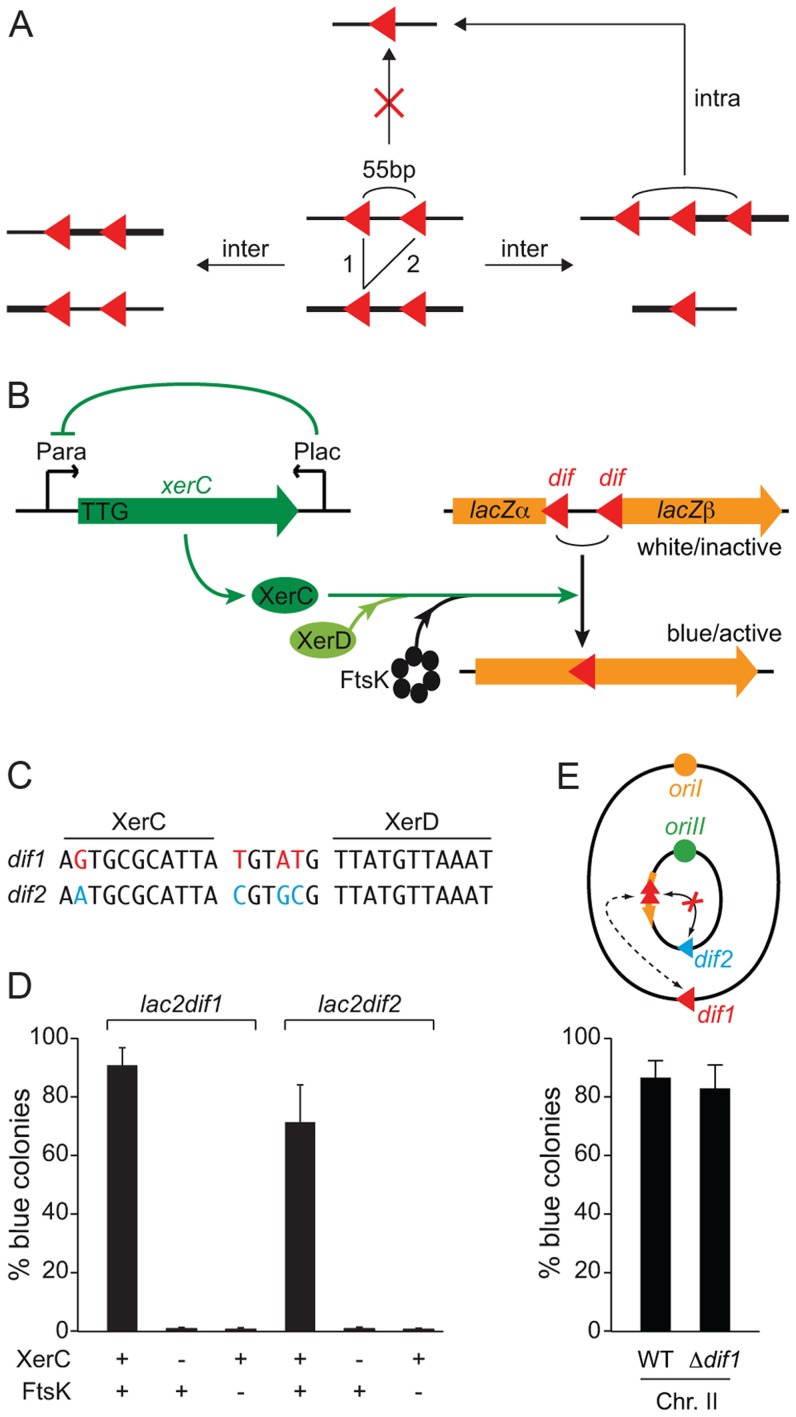
FtsK-dependent Xer recombination at *dif* as a tool to monitor sister chromatid contacts. **A.** Scheme of the different site-specific recombination products that can be obtained when intramolecular recombination is impeded. **B.** Schematic representation of the 55 bp *dif*-recombination cassettes and the Xer recombination control set up. The orange arrow represents the *lacZ* gene disrupted by the two *dif* sites (red triangle). Expression of the *xerC* gene (green arrow) is under the positive control of the arabinose promoter, P_BAD_, and the negative control of the *E. coli lacZ* promoter, P_Lac_. **C.** *dif1* and *dif2* sequence divergence. The DNA binding arms of XerC and XerD are shown. Bases that differ in *dif1* and *dif2* are shown in red and blue, respectively. **D.** Reconstitution of a functional *lacZ* gene depends on XerC and FtsK. Results from at least three independent experiments. **E.** Recombination between *dif* sites harboured on different chromosomes does not perturb the SCC detection. Schematic representation of the genome of a strain harbouring *lac2dif1* on chrII. No intrachromosomal recombination can occur between *lac2dif1* and *dif2* because of sequence divergence. The influence of chrI *dif1* on chrII *lac2dif1* recombination was tested by comparing results obtained in a strain in which *dif1* was deleted. Results from at least three independent experiments. Legends as in Figure 1A. Red triangle: *dif1*; blue triangle: *dif2*.

As the densities of *matS* sites in TerI and TerII are very similar, we were intrigued by the apparent inability of MatP to block TerII sister separation. Lesterlin *et al.* designed an assay based on the interruption of the *lacZ* reporter gene by two copies of *loxP* to detect sister chromatid contacts (SCC) behind replication forks [33]. The assay was based on the proximity of the *loxP* sites: the cleavage points of the Cre recombinases on each strand of the tandem sites were separated by only 55 bp to prevent intra-molecular recombination. As a result, a functional *lacZ* ORF could only be reconstructed via intermolecular recombination events (Figure 2A). As *dif*-recombination is under the control of FtsK in *V. cholerae* [26], which was expected to restrict it to mid-cell and to the time of septum constriction [21], we reasoned that 55 bp *dif*-cassettes could be used to monitor the proximity of TerI and TerII sisters to the cell division machinery at the time of constriction (Figure 2B).

We engineered a strain in which XerC production was under the control of the arabinose promoter to permit the stable inheritance of *dif*-cassettes. To help repress any leaky XerC production, we inserted the *E. coli lacZ* promoter and the *E. coli lacI* repressor gene in anti-orientation at the end of the *xerC* ORF. We also replaced the ATG translation initiation codon by the less favourable TTG codon and removed the ribosomal binding site (Figure 2B).

The *dif* sites harboured by the first and second chromosomes of the El Tor N16961 strain, *dif*1 and *dif*2, possess divergent overlap regions (Figure 2C, [26], [34]). To compare the excision of 55 bp *dif1*- and *dif2*-cassettes (*lac2dif1* and *lac2dif2*), we inserted them at the same genomic position, in place of the *dif* locus of chromosome II, and monitored the frequency of full blue colonies that were obtained three hours after the induction of XerC production (Figure 2C). Recombination worked well for both *dif* sites (Figure 2D). In both cases, blue colony formation strictly depended on XerC production and on the presence of a fully functional *ftsK* allele (Figure 2D).

Little or no recombination can occur between *dif1* and *dif2* thanks to their sequence divergence (Figure 2C). The use of *lac2dif2* on chrI and *lac2dif1* on chrII thus prevented any risk of Xer-mediated intrachromosomal rearrangements due to recombination between the *dif* sites of the cassette and the dimer resolution site of the chromosome during the course of the experiment (Figure 2E). Therefore, the dimer resolution site of the chromosome could be left, which avoided any artefact in the measured excision frequencies linked to the formation of chromosome dimers by recombination between sister copies of the cassettes (Figure S5). The *dif* sites of the cassettes used on each of the two *V. cholerae* chromosomes are identical to the dimer resolution site of the other chromosome. However, this site did not influence the proportion of blue colonies that were formed (Figure 2E and Figure S6).

**Figure 3:**
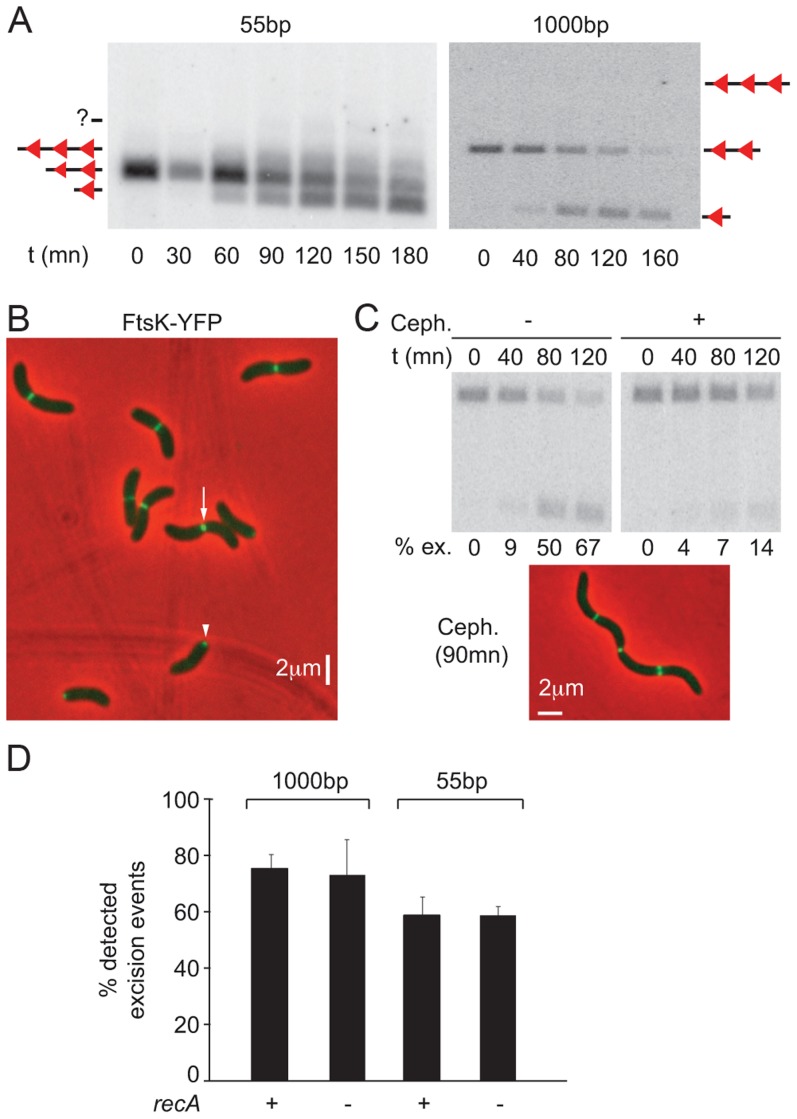
Control of *dif*-recombination. **A.** Structural control of Xer recombination. Southern blot showing the different recombination products obtained with 55 bp- and 1 kbp- cassettes inserted at the *dif1* locus. **B.** FtsK-YFP localization. The white arrow indicates a cell in which FtsK is located at the septum; the white arrowhead shows a cell in which FtsK is located at the new pole. **C.** Temporal control of Xer recombination. Upper panels: southern blot showing the excision of a 1 kbp cassette inserted at the *dif1* locus, without or with cephalexin treatment. t: time of the experiment; ex.: excision frequency. Lower panel: snapshot showing that cephalexin treatment results in filamentation but does not prevent FtsK localization to mid-cell. **D.** RecA-independent recombination between *dif2* sites inserted at the *dif1* locus.

Both intramolecular and intermolecular recombination events can generate single *dif* site products. In contrast, three *dif* site products can only be generated via intermolecular recombination. Such products are transient because they can be converted to single *dif* products by subsequent intramolecular recombination (Figure 2A). Nevertheless, we could detect their appearance with 55 bp cassettes, demonstrating that recombination occurred via SCC (Figure 3A). As a point of comparison, we engineered 1 kbp *dif*-cassettes, a distance sufficient for intramolecular recombination. With such cassettes, we did not observe any intermolecular recombination intermediates, suggesting that 1 kbp cassette excision mainly resulted from intramolecular recombination events on separate chromatids (Figure 3A).

FtsK-YFP localized to mid-cell in long cells (Figure 3B, white arrow) and at one of the two poles in short cells (Figure 3B, white arrow head). This was reminiscent of the pattern of localization of the cell division machinery of *Caulobacter crescentus*, which assembles at mid-cell but remains bound to the new pole after cell scission [35]. Time-lapse observations confirmed that such a scenario applied to *V. cholerae* FtsK, demonstrating that it assembled at mid-cell as part of the cell division machinery (Figure S7A). In addition, treating cells with cephalexin, which blocks septum constriction, led to a dramatic reduction in the level of *dif*-recombination without affecting the recruitment of FtsK to the cell division apparatus (Figure 3C). No loss of cell viability was observed during the course of the cephalexin treatment (Figure S7B). We conclude that *dif*-recombination occurs during or shortly after septum constriction in *V. cholerae*.

Finally, deletion of *recA* did not affect the proportion of excision events that could be detected using 55 bp- and 1 kbp-cassettes, indicating that activation of *dif*-recombination was independent from chromosome dimer formation in *V. cholerae* (Figure 3D). This result is strikingly different from what is observed using *dif*-cassettes in *E. coli* [36], [37]. The reasons for this difference are the subject of another study (Gally, Midonet, Demarre and Barre, unpublished results).

Taken together, these results demonstrate that the proportion of blue colonies formed following *lac2dif1* and *lac2dif2* recombination events can be used as a relative measure of the respective frequency of contacts between monomeric sister chromatids that occur at mid-cell at the time of septum constriction in *V. cholerae*.

### High frequency of TerI and TerII SCC during septum constriction

**Figure 4:**
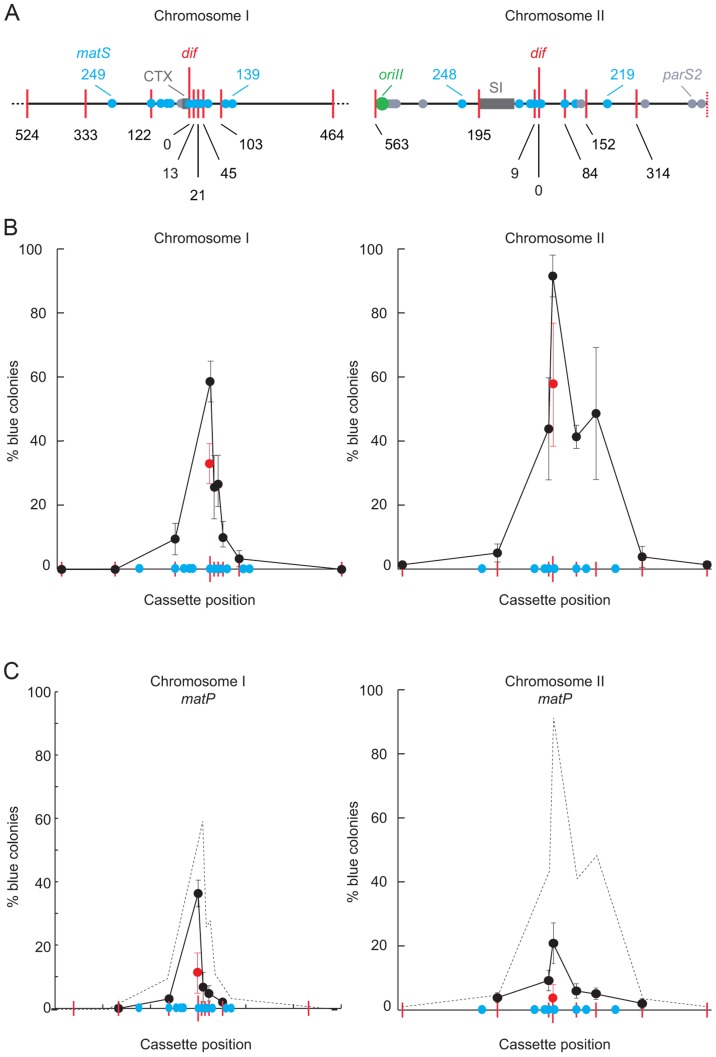
Influence of MatP on SCC during septation. **A.** *V. cholerae* chrI and chrII maps centred on their dif locus. Red bars: positions of *lac2dif* cassettes; blue disks: positions of *matS* sites; light grey disks: positions of *parS2* sites; dark grey rectangle: recent chromosomal insertions (CTX: Integrative Mobile Elements Exploiting Xer; SI: superintegron). Only 1 Mbp of chrI is depicted. **B.** Relative frequency of SCC during septation as a function of the position of the distance from the *dif* locus (in kbp). Results from at least three independent experiments. **C.** Influence of MatP on sister chromatid contacts. Results from at least three independent experiments. The dashed grey curves show the results obtained in B.

Cells in which *lac2dif2* were inserted in the immediate vicinity of *dif1* yielded a high level (∼60%) of blue colonies, demonstrating *dif1* SCC during constriction (Figure 4A), in agreement with the co-localization of *dif1* sisters (Figure 1). However, interchromatid recombination dropped rapidly when *lac2dif2* was not in the immediate vicinity of the *dif1* locus (Figure 4A). The frequency of blue colony formation did not diminish in cells in which *recA* was deleted, confirming that 55 bp cassette recombination on chrI was not restricted to chromosome dimers (Figure S8A).

Strikingly, we obtained a very high proportion of blue colonies (∼90%) when *lac2dif1* was inserted at *dif2* (Figure 4B) despite the apparent early separation of *dif2* sisters (Figure 1). In addition, blue colony formation remained high (∼45%) within a 160 kbp region surrounding *dif2*, from a position at 9 kb on the left of the *dif* locus to 152 kb on the right of it (Figure 4B). The same results were obtained after *recA* deletion, confirming that TerII SCCs were unlikely due to chromosome dimers (Figure S8A). Taken together those results suggested that *dif2* sisters contacted each other at mid-cell at the time of cell division as frequently as *dif1* sisters, despite their apparent early separation.

### MatP drives TerII sister contacts during septum constriction

On chrII, the extent of the region displaying a high frequency of SCC at the time of septum constriction corresponded to the putative MatP domain (Figure 4B). The only notable exception was next to a *matS* site that is isolated from the rest of the *matS* region by the *V. cholerae* superintegron (Figure 4B). Correspondingly, we observed more than a 4-fold reduction in blue colony formation within TerII upon *matP* disruption (Figure 4C). Indeed, *dif2* was the only locus where cassette excision remained above the background level (Figure 4C). Cassette excision remained independent from chromosome dimer formation (Figure S8B). In contrast, the disruption of *matP* only had a very modest, albeit significant, effect on SCCs within TerI (Figure 4C). The remaining SCCs were still independent from homologous recombination (Figure S8B). Correspondingly, SCCs occurred in a much smaller region than the putative MatP domain on chrI (Figure 4B). Taken together, these results suggested that MatP was the main contributor to TerII SCC occurring at mid-cell at the time of cell division.

### Direct time-lapse observation of TerII SCC during cell division

The high frequency of SCCs detected at dif2 with our genetic assay suggested that *dif2* sisters frequently collided at mid-cell during septum constriction despite their early separation. To directly demonstrate that such collisions occurred, we followed the segregation dynamics of *dif2* sisters by time-lapse fluorescence microscopy. We expected collisions to be transient because two *dif2* spots were observed in almost all of the wild type cells longer than 4.5 µm (Figure 1 and Figure S9A). Therefore, we reasoned that short time intervals had to be used between each image acquisition. However, a balance had to be achieved between the detection of the supposedly transient *dif2* collisions and the fraction of the cell cycle during which *dif2* spots could be tracked in any given cell due to photobleaching. With 30 s time intervals, *dif2* foci could be observed for 100 min.

**Figure 5:**
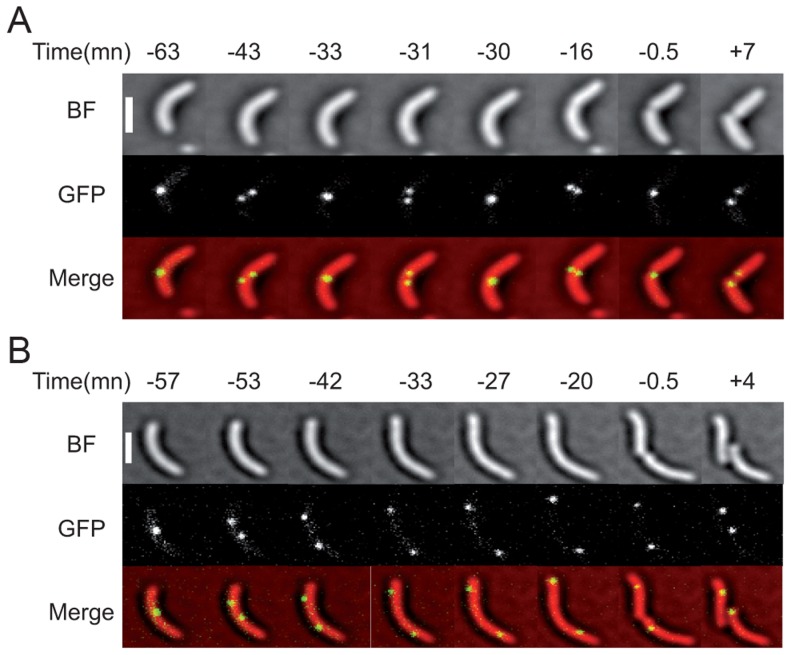
Two examples of the *dif2* choreography observed by video microscopy. (A) WT cells. (B) *matP* ^−^ cells. The time before or after the cell division event is indicated in minutes. The phase images obtained from the BF images, the fluorescence images and the merge are shown. The scale bar corresponds to 2 µm. Other examples are presented in Movie S1 and 2.

A total of 74 wild-type cells were followed, out of which 44 showed a complete cell division event. In 42 of these cells, i.e. in ∼95% of the observed cell division events, *dif2* sisters separated before septum invagination, in agreement with our snapshot analysis. However, *dif2* sister collisions were frequent (Figure 5A and Movie S1). As a result, *dif*2 sisters were found to co-localize at mid-cell at some stage of the cell constriction process in 70% of the cells, which fits with the high frequency of *dif2* SCCs observed with the genetic assay (Figure 5A and Movie S1). On average, 3.2 collisions were observed after the initial separation of the *dif2* sisters and before cell fission. In the majority of cases, re-joining of the *dif2* sisters was transient, i.e. co-localization was only observed during 2 consecutive frames. In some instances, however, *dif2* sisters remained co-localized for several minutes.

We also followed 131 *matP^−^* cells, out of which 30 displayed a complete analysable cell division event. In all of these cells, *dif2* sisters separated before septum invagination (Figure 5A and Movie S2). The positions of the two *dif2* sisters were no longer restricted to the ¼–¾ cell region and, in several cases, one of the two *dif2* spots located near the old pole at the time of division (Figure 5A and Movie S2). Indeed, only 0.6 collisions were observed on average in each cell after the initial separation of the *dif2* sisters and before cell fission. These events lasted for a single frame in the vast majority of cases. Finally, co-localization of the *dif2* sisters during septum constriction was only observed once, which fits with the loss of *dif2* SCCs monitored with the genetic assay.

Taken together, these results suggested that MatP allowed FtsK to process *dif2* sisters during cell division by restricting the range of their movements to the ¼–¾ cell region and that other factors played a similar role for *dif1* sisters in its absence.

## Discussion

In the present study, we investigated the positioning of the replication terminus regions of the two *V. cholerae* chromosomes using a combination of two techniques. On one hand, we directly visualized the positioning of the chromosome dimer resolution locus of each of the two chromosomes by snapshot and time-lapse microscopy (Figure 1 and Figure 5). On the other hand, we monitored the proximity of these loci using Xer recombination between sister *dif* sites as a genetic reporter (Figure 4). The requirement for a direct contact between the Xer recombinases and the FtsK cell division protein for recombination to occur ensured that sister sites were recombined at mid-cell (Figure 2 and 3). The requirement for constriction initiation ensured that they were recombined at the time of septum constriction (Figure 3). The high frequency of intermolecular recombination events at the chromosome I *dif* locus (Figure 4) could be due to the late separation of the two sister chromatids at this particular location (Figure 1 and Figure 5). Intermolecular recombination events at the chromosome II *dif* locus could also happen before the sisters segregated, i.e. after *dif* duplication but before replication completion and/or when separated sisters were still trapped together by catenation links. However, this can only account for a limited of number of recombination events since snapshot analysis and time-lapse microscopy suggested that sisters of the chromosome II *dif* locus separated before septum constriction in ∼95% of the dividing cells (Figure 1 and Figure 5). Thus, the high frequency of recombination events between chromosome II *dif* sisters is probably mainly linked to collisions events that occurred their initial separation (Figure 4).

### MatP-dependent coordination of TerII segregation with cell division

Possibly the most striking observation of our study was that TerII sisters kept colliding against each other at mid-cell after their initial separation in the cell cycle, up to and after the initiation of the constriction process (Figure 4 and 5). During the three hours of our genetic assays, cells underwent ∼8 divisions, as judged by the number of colony forming units at the beginning and at the end of the experiments. Therefore, the ∼90% frequency of blue colony formation that we observed with a recombination inserted at *dif2* corresponded to a rate of 25% of β-galactosidase^+^ cell formation per generation. As only one out of the two possible intermolecular recombination events could yield β-galactosidase+ cells (Figure 2A), this result suggested that >50% of SCC occurred between TerII sisters during each cell division event (Figure 4). Moreover, we observed the same frequency of blue colony formation with the *lacZdif2* probe when it was inserted at the *dif2* locus on chrII (Figure 2D, *lac2dif2*) and when it was inserted at the *dif1* locus on chrI (Figure 4B, *dif1* locus), suggesting that SCCs at cell division were as frequent within TerII as within TerI. Accordingly, frequent collisions of *dif2* sisters were observed at the time of cell division when following the growth of individual cells by fluorescence microscopy with 30 s time intervals (Figure 5). Interchromatid recombination events during constriction were observed in a specific 160 kb region of chrII, which corresponded to the putative MatP domain of the chromosome (Figure 4). The relative frequency of interchromatid recombination curve consisted of a plateau with a central peak at the *dif2* locus (Figure 4). Our results suggested the plateau was due to the action of the MatP/*matS* system (Figure 4).

### Management of TerI during cell division

Our snapshot analysis of the positioning of *dif1* in wild type and *matP^−^* cells indicated that MatP was a major contributor to the organization and management of TerI at the time of cell division, as observed in *E. coli* (Figure 1). However, the relative frequency of interchromatid recombination curve on chrI simply consisted of a sharp peak centred on *dif1* with no plateau in the MatP region (Figure 4). In addition, the relative frequency of SCCs was not dramatically affected in *matP^−^* cells (Figure 4). This is in sharp contrast to what we could have expected based on the role of MatP in the formation of a FtsK loading region in *E. coli* [13]. Taken together, these observations suggest that other factors than MatP contribute to the management of *dif1* sisters at the time of cell division, which partially masked its action in our genetic assay. We are currently investigating the relative contribution of likely candidates for TerI mid-cell localization using the power of our SCC assay. We think that these factors might be common to other bacteria in which sister copies of the terminus regions remain at mid-cell for a long period during cell division, such as *P. aeruginosa* and *C. crescentus*. However, they could not, or might not yet, be adapted to the management of the recently acquired chrII of *V. cholerae*. As a result, the MatP/*matS* system was left as the sole contributor for TerII SCCs during cell division, which helped reveal its action.

### MatP mechanism of Ter organization

The disruption of *matP* had a profound impact on the subcellular localization of *dif1* and *dif2* (Figure 1). In particular, MatP seemed to impede the separation of *dif1* sisters until cell division (Figure 1). MatP is able to create bridges between two *matS* sites [38]. However, we do not think that sister chromatids are tethered together by such bridges since MatP did not impede the separation of *dif2* sisters (Figure 1). Careful analysis of the location of *dif1* and *dif2* spots in wild type and *matP^−^* cells rather suggested that MatP helped create a molecular leash that confined Ter regions in the ¼–¾ portion of the cell: even though the median positions of *dif2* sisters in the cell population indicated their separation before cell division, they did not migrate very far apart from each other and away from mid-cell (Figure 1). In particular, ∼90% of *dif2* spots were located at a distance of less than a quarter of the cell length in cells longer than 4.5 µm (Figure S9A). Results from our genetic assay suggested that the movements of such sister loci around the median position probably allowed for their frequent collision at mid-cell at the time of cell division. Even though their medians were equivalent, the distributions of the distances between *dif2* sisters in wild type and *matP^−^* cells were markedly different (Figure 1G). Indeed, in *matP^−^* cells longer than 4.5 µm, only ∼57% of the *dif2* spots remained in the ¼–¾ portion of the cell (Figure S9B). This might be sufficient to explain a large drop in sister collisions. In contrast, ∼83% of *dif1* spots remained in the ¼–¾ portion of the cell, which might explain the low impact of the *matP* disruption on the frequency of SCC (Figure S9C and S9D). Further work will be required to investigate the molecular nature of the MatP leash. An attractive possibility would be that MatP restrains the movement of catenation loops between the two circular chromatid sisters by binding together the *matS* sites of each sister chromatid.

### Ter positioning as a key feature of the bacterial cell cycle

Our results suggest that multiple redundant factors, including MatP in the *enterobacteriaceae* and the Vibrios, ensure that sister copies of the terminus region of bacterial chromosomes remain sufficiently close to mid-cell to be processed by FtsK. In this regard, it is remarkable to observe that, even though initiation of chrII replication responds to the same global cell cycle regulatory networks than chrI initiation [39], it occurs at a later time point in the cell cycle [28], which results in synchronous chrI and chrII replication termination (Figure S1, [28]). This is likely to participate in delaying TerII sister separation until the time of cell division. We observed that *matP^−^* cells were longer than wild type cells in agreement with the notion that coordination of cell division and chromosome segregation is a key feature of the bacterial cell cycle (Figure S1). What is the functional role of this coordination? The late segregation of the terminus region might facilitate the action of FtsK on unresolved catenation links or chromosome dimers. Under laboratory conditions, we did not observe any significant chromosome dimer resolution defect (Figure S4). However, these results have to be interpreted with caution since the disorganization induced by the absence of MatP should only slightly delay the time required for FtsK to bring together sister *dif* sites.

## Methods

### Strains and plasmids

Genetic engineering methods are described in Text S1. Bacterial strains and plasmids used in this study are listed in Tables S1 and S2, respectively. All *V. cholerae* strains were derivatives of the El Tor N16961 strain.

### Fluorescent microscopy observations

A *lacO* array was inserted adjacent to *dif1* and a PMT1 *parS* was inserted adjacent to *dif2*. LacI^E.coli^-mCherry and yGFP-ParB^pMT1^ were produced via the leaky expression of a synthetic operon under the *E. coli* lacZ promoter that was inserted at the *V. cholerae lacZ* locus. A C-terminal fusion between FtsK and a yellow fluorescent protein, FtsK-YFP, was inserted in place of the endogenous *V. cholerae ftsK* allele to visualize its localisation. Protocols for Microscopy are detailed in Text S1. The snapshot images were analysed using the Matlab-based sofware MicrobeTracker [40], [41]. Details for the analysis are described in [27].

### Time-lapse fluorescent microscopy

For bright field (BF) and fluorescence microscopy 2 µl of an exponentially growing culture sample were placed on a microscope slide coated with a thin agarose layer (1%) made using the growth medium. The slide was incubated at 30°C during the images acquisition. The images were acquired with an Evolve 512 EMCCD camera attached to an Axio Observe spinning disk from Zeiss and recorded every 30 seconds with step size of 0.4 µm in the Z-axis (3 images were acquired for each channel). The BF image 3 is subtracted from the BF image 1 to obtain the phase image.

### *dif*-recombination assays

Blue colony formation assay: 0.2 mM IPTG were used to repress *xerC* transcription. 0.1% arabinose was used to produce XerC. Freshly grown cultures were diluted in 5 mL of LB supplemented with arabinose to reach 0.02 of optical density at 600 nm. They were incubated for 180 mn at 37°C with shaking. Serial dilutions of the cells were plated on LB agar plates supplemented with X-gal and IPTG before and after the induction of recombination.

Southern blot assay: Cephalexin was added at the final concentration of 10 µg/ml at the same time as the arabinose. Cells were collected at the beginning of the incubation and after 40, 80 and 120 mn for genomic DNA extraction. Recombination products were analysed an EcoRV/HphI digest and 1 kbp fragment corresponding to the *lacZ* promoter as a probe. Signals were detected using a Typhoon instrument and quantified using the IQT 7.0 software (GE Healthcare).

## Supporting Information

Figure S1 Replication profiling of the two *V. cholerae* chromosomes in WT (**A**) and *matP^−^* (**B**) cells. To compare the timing of replication of chrI and chrII in the two genetic backgrounds, we calculated the relative frequency of uniquely mapping sequence tags within the genomic DNA of exponentially growing (replicating) cells in 1000 bp windows, i.e. the number of uniquely mapping sequence tags within each 1000 bp window divided by the total number of sequence that were uniquely mapped in the entire genome [42]. Left panels: chrI replication profiling; Right panels: chrII replication profiling. The position of the terminus is indicated by two facing red triangles. A yellow sun indicates the location of the single locus that had been so far visualized in the Ter of each of the two chromosomes.
(PDF)

Figure S2 Cell distribution in the WT strain (**A**) of *V. cholerae* or its *matP^−^* mutant (**B**). Cells were classified according to their length in bins of 0.25 µm. The dashed line shows the limit of 30 cells under which data was plotted in grey elsewhere in the manuscript.
(PDF)

Figure S3 Differences in the pattern of segregation of Ter I and Ter II are not due to the fluorescent microscopy visualization tools. The fluorescent markers that were used in Fig. 1 to label the *dif1* and *dif2* loci were switched: the *dif1* locus was visualized using the YGFP–ParB^PMT1^/*parS* system and the *dif2* locus was visualized with the *lacO*/LacI system. **A.** Relative position of *dif1* (in black) and *dif2* (in red) along the long axis of the cell as a function of cell length. **B.** Frequency of cells with separated *dif1* (in black) and *dif2* (in red) sisters as a function of cell length. The plain red and black lines show the data for the bins containing at least 30 cells; the dashed grey lines show the data for bins containing 3 to 29 cells. **C.** Interfocal distance of the sister copies of the *dif* locus of each of the two *V. cholerae* chromosomes, (*dif1* in black and *dif2* in red). **D.** Cell distribution. Cells were classified according to their length in bins of 0.25 µm. The dashed line shows the limit of 30 cells under which data was plotted.
(PDF)

Figure S4 Graphic representation of growth competition between mutant strains of *V. cholerae* and a WT strain. The ratio of the mutant against its parental strain is plotted as a function of the number of generation. Cells were grown in parallel at 30°C with a 10^−4^ or a 10^−5^ dilution every 12 h for 5 days. Cell dilutions were plated every 24 h on cognate antibiotic plates to determine the number of CFU of the mutant versus the WT strain. The generation time between every time point was calculated from these numbers. The CFU ratio between mutant and its parental strain varies with the number of generations and it can be used to determine the loss of fitness of every mutant. The fitness loss for *matP^−^* cells was ≅0.23% (blue), for Δ*dif1* cells it was ≅6.9% (red), for Δ*dif1 matP^−^* it was ≅5.9% (orange), for Δ*dif2* it was ≅2% (green) and for Δ*dif2 matP^−^* it was ≅1.5 (yellow).
(PDF)

Figure S5 Schematic representation of the possible intermolecular recombination events between *lac2dif1* cassettes harboured on TerII sister chromatids. Green dot: oriII. Blue triangle: *dif2*. Red triangle: *dif1*. The orange arrow represents the *lacZ* gene disrupted by the two *dif1* sites (*lac2dif1*) or three *dif1* sites (*lac3dif1*). The blue arrows show the functional lacZ gene after the deletion of one *dif1* site (*lac1dif1*).
(PDF)

Figure S6 Recombination between *dif* sites harboured on different chromosomes does not perturb the SCC detection. Schematic representation of the genome of a strain harbouring *lac2dif2* on chI. No intrachromosomal recombination can occur between *lac2dif2* and *dif1* because of sequence incompatibility. The influence of chII *dif2* on chI *lac2dif2* recombination was tested by comparing results obtained in a strain in which *dif2* was deleted. Results from at least three independent experiments. *oriI* represented with an orange dot and *oriII* by a green dot. *dif1* is represented by a red triangle and *dif2* with a blue triangle, the orange arrow show the *lacZ* gene disrupted by the two *dif1* sites.
(PDF)

Figure S7 (**A**) FtsK targets to midcell prior to cell division. Localization of FtsK-YFP in cells seen by video microscopy. The time before or after the first cell division event is indicated in minutes. (**B**) 2 h cephalexin treatment does not affect *V. cholerae* survival. Cells were grown without (plain line) or with (dashed line) cephalexin and spread on LB agar plates for cfu determination every 40 min. When cells were treated with cephalexin, the number of cfu didn't increase (as expected since cells can't divide) but remained constant.
(PDF)

Figure S8 Dimer formation does not influence SCC in *V. cholerae*. (**A**) Dimer formation does not influence SCC at *dif2* in a WT background. The same is true at *dif1* (Figure 3D). (**B**) Dimer formation does not influence SCC at *dif1* and *dif2* in a *matP^−^* background. Results from at least three independent experiments.
(PDF)

Figure S9 Localization of *dif1* and *dif2* in WT and *matP^−^* strains of *V. cholerae* **.** *dif1* (**A** and **C**) and *dif2* (**B** and **D**) were localized in exponentially growing WT cells *(GDV552;* ***A*** *and* ***B*** *)* and *matP^−^* cells (GDV564, **C** and **D**). The localization was done using the lacO/lacI-mcherry system at *dif1* and YGFP–ParB^PMT1^/*parS* system at *dif2*. Plots show focus relative positions in cells with one (red) focus, and two (blue and black) foci as a function of the cell length.
(PDF)

Movie S1 Choreography of *dif2* sisters in WT cells seen by video-microscopy. Cells were grown on a thin agarose layer (1%) at 30°C. Images were acquired every 30 seconds. Movies are shown at a rate of 4 frames/sec.
(MP4)

Movie S2 Choreography of *dif2* sisters in *matP^−^* cells seen by video-microscopy. Cells were grown on a thin agarose layer (1%) at 30°C. Images were acquired every 30 seconds. Movies are shown at a rate of 4 frames/sec.
(MP4)

Table S1 List of bacterial strains.
(DOCX)

Table S2 List of plasmids.
(DOCX)

Text S1 Genetic engineering and microscopic analysis methods.
(DOCX)

## References

[1] Egan ES, Fogel MA, Waldor MK (2005) Divided genomes: negotiating the cell cycle in prokaryotes with multiple chromosomes. Mol Microbiol 56: 1129–1138. 15882408 10.1111/j.1365-2958.2005.04622.x

[2] Trucksis M, Michalski J, Deng YK, Kaper JB (1998) The Vibrio cholerae genome contains two unique circular chromosomes. Proc Natl Acad Sci U S A 95: 14464–14469. 9826723 10.1073/pnas.95.24.14464 PMC24396

[3] Xu Q, Dziejman M, Mekalanos JJ (2003) Determination of the transcriptome of Vibrio cholerae during intraintestinal growth and midexponential phase in vitro. Proc Natl Acad Sci U S A 100: 1286–1291. 12552086 10.1073/pnas.0337479100 PMC298765

[4] Rosa PA, Tilly K, Stewart PE (2005) The burgeoning molecular genetics of the Lyme disease spirochaete. Nat Rev Microbiol 3: 129–143. 15685224 10.1038/nrmicro1086

[5] Holden MT, Titball RW, Peacock SJ, Cerdeno-Tarraga AM, Atkins T, et al (2004) Genomic plasticity of the causative agent of melioidosis, Burkholderia pseudomallei. Proc Natl Acad Sci U S A 101: 14240–14245. 15377794 10.1073/pnas.0403302101 PMC521101

[6] Casjens S (1998) The diverse and dynamic structure of bacterial genomes. Annu Rev Genet 32: 339–377. 9928484 10.1146/annurev.genet.32.1.339

[7] Thompson FL, Iida T, Swings J (2004) Biodiversity of vibrios. Microbiol Mol Biol Rev 68: 403–431 table of contents. 15353563 10.1128/MMBR.68.3.403-431.2004 PMC515257

[8] Kono N, Arakawa K, Tomita M (2011) Comprehensive prediction of chromosome dimer resolution sites in bacterial genomes. BMC Genomics 12: 19. 21223577 10.1186/1471-2164-12-19 PMC3025954

[9] Possoz C, Junier I, Espeli O (2012) Bacterial chromosome segregation. Front Biosci (Landmark Ed) 17: 1020–1034. 22201788 10.2741/3971

[10] Vallet-Gely I, Boccard F (2013) Chromosomal organization and segregation in Pseudomonas aeruginosa. PLoS Genet 9: e1003492. 23658532 10.1371/journal.pgen.1003492 PMC3642087

[11] Harms A, Treuner-Lange A, Schumacher D, Sogaard-Andersen L (2013) Tracking of Chromosome and Replisome Dynamics in Myxococcus xanthus Reveals a Novel Chromosome Arrangement. PLoS Genet 9: e1003802. 24068967 10.1371/journal.pgen.1003802 PMC3778016

[12] Joshi MC, Bourniquel A, Fisher J, Ho BT, Magnan D, et al (2011) Escherichia coli sister chromosome separation includes an abrupt global transition with concomitant release of late-splitting intersister snaps. Proc Natl Acad Sci U S A 108: 2765–2770. 21282646 10.1073/pnas.1019593108 PMC3041144

[13] Stouf M, Meile JC, Cornet F (2013) FtsK actively segregates sister chromosomes in Escherichia coli. Proc Natl Acad Sci U S A 110: 11157–11162. 23781109 10.1073/pnas.1304080110 PMC3704039

[14] Thiel A, Valens M, Vallet-Gely I, Espeli O, Boccard F (2012) Long-range chromosome organization in E. coli: a site-specific system isolates the Ter macrodomain. PLoS Genet 8: e1002672. 22532809 10.1371/journal.pgen.1002672 PMC3330122

[15] de Boer PA (2010) Advances in understanding E. coli cell fission. Curr Opin Microbiol 13: 730–737. 20943430 10.1016/j.mib.2010.09.015 PMC2994968

[16] Wu LJ, Errington J (2004) Coordination of cell division and chromosome segregation by a nucleoid occlusion protein in Bacillus subtilis. Cell 117: 915–925. 15210112 10.1016/j.cell.2004.06.002

[17] Espeli O, Borne R, Dupaigne P, Thiel A, Gigant E, et al (2012) A MatP-divisome interaction coordinates chromosome segregation with cell division in E. coli. EMBO J 31: 3198–3211. 22580828 10.1038/emboj.2012.128 PMC3400007

[18] Mercier R, Petit MA, Schbath S, Robin S, El Karoui M, et al (2008) The MatP/matS site-specific system organizes the terminus region of the E. coli chromosome into a macrodomain. Cell 135: 475–485. 18984159 10.1016/j.cell.2008.08.031

[19] Dubarry N, Barre FX (2010) Fully efficient chromosome dimer resolution in Escherichia coli cells lacking the integral membrane domain of FtsK. EMBO J 29: 597–605. 20033058 10.1038/emboj.2009.381 PMC2830691

[20] Saleh OA, Perals C, Barre FX, Allemand JF (2004) Fast, DNA-sequence independent translocation by FtsK in a single-molecule experiment. EMBO J 23: 2430–2439. 15167891 10.1038/sj.emboj.7600242 PMC423284

[21] Kennedy SP, Chevalier F, Barre FX (2008) Delayed activation of Xer recombination at dif by FtsK during septum assembly in Escherichia coli. Mol Microbiol 68: 1018–1028. 18363794 10.1111/j.1365-2958.2008.06212.x

[22] Espeli O, Lee C, Marians KJ (2003) A physical and functional interaction between Escherichia coli FtsK and topoisomerase IV. J Biol Chem 278: 44639–44644. 12939258 10.1074/jbc.M308926200

[23] Bigot S, Marians KJ (2010) DNA chirality-dependent stimulation of topoisomerase IV activity by the C-terminal AAA+ domain of FtsK. Nucleic Acids Res 38: 3031–3040. 20081205 10.1093/nar/gkp1243 PMC2875013

[24] Lesterlin C, Pages C, Dubarry N, Dasgupta S, Cornet F (2008) Asymmetry of chromosome Replichores renders the DNA translocase activity of FtsK essential for cell division and cell shape maintenance in Escherichia coli. PLoS Genet 4: e1000288. 19057667 10.1371/journal.pgen.1000288 PMC2585057

[25] Dubarry N, Possoz C, Barre FX (2010) Multiple regions along the Escherichia coli FtsK protein are implicated in cell division. Mol Mic 78: 1088–1100. 10.1111/j.1365-2958.2010.07412.x 21091498

[26] Val M-E, Kennedy SP, El karoui M, Bonné L, Chevalier F, et al (2008) FtsK-dependent dimer resolution on multiple chromosomes in the pathogen Vibrio cholerae. PLoS Genet 4: e1000201. 18818731 10.1371/journal.pgen.1000201 PMC2533119

[27] David A, Demarre G, Muresan L, Paly E, Barre FX, et al (2014) The two Cis-acting sites, parS1 and oriC1, contribute to the longitudinal organisation of Vibrio cholerae chromosome I. PLoS Genet 10: e1004448. 25010199 10.1371/journal.pgen.1004448 PMC4091711

[28] Rasmussen T, Jensen RB, Skovgaard O (2007) The two chromosomes of Vibrio cholerae are initiated at different time points in the cell cycle. Embo J 26: 3124–3131. 17557077 10.1038/sj.emboj.7601747 PMC1914095

[29] Yamaichi Y, Bruckner R, Ringgaard S, Moll A, Cameron DE, et al (2012) A multidomain hub anchors the chromosome segregation and chemotactic machinery to the bacterial pole. Genes Dev 26: 2348–2360. 23070816 10.1101/gad.199869.112 PMC3475806

[30] Yamaichi Y, Fogel MA, Waldor MK (2007) par genes and the pathology of chromosome loss in Vibrio cholerae. Proc Natl Acad Sci U S A 104: 630–635. 17197419 10.1073/pnas.0608341104 PMC1760642

[31] Brezellec P, Hoebeke M, Hiet MS, Pasek S, Ferat JL (2006) DomainSieve: a protein domain-based screen that led to the identification of dam-associated genes with potential link to DNA maintenance. Bioinformatics 22: 1935–1941. 16787973 10.1093/bioinformatics/btl336

[32] Srivastava P, Fekete RA, Chattoraj DK (2006) Segregation of the replication terminus of the two Vibrio cholerae chromosomes. J Bacteriol 188: 1060–1070. 16428410 10.1128/JB.188.3.1060-1070.2006 PMC1347332

[33] Lesterlin C, Gigant E, Boccard F, Espeli O (2012) Sister chromatid interactions in bacteria revealed by a site-specific recombination assay. EMBO J 31: 3468–3479. 22820946 10.1038/emboj.2012.194 PMC3419930

[34] Das B, Bischerour J, Val M-E, Barre FX (2010) Molecular keys of the tropism of integration of the cholera toxin phage. PNAS 107: 4377–4382. 20133778 10.1073/pnas.0910212107 PMC2840090

[35] Goley ED, Yeh YC, Hong SH, Fero MJ, Abeliuk E, et al (2011) Assembly of the Caulobacter cell division machine. Mol Microbiol 80: 1680–1698. 21542856 10.1111/j.1365-2958.2011.07677.x PMC3707389

[36] Perals K, Capiaux H, Vincourt JB, Louarn JM, Sherratt DJ, et al (2001) Interplay between recombination, cell division and chromosome structure during chromosome dimer resolution in Escherichia coli. Mol Microbiol 39: 904–913. 11251811 10.1046/j.1365-2958.2001.02277.x

[37] Barre FX, Aroyo M, Colloms SD, Helfrich A, Cornet F, et al (2000) FtsK functions in the processing of a Holliday junction intermediate during bacterial chromosome segregation. Genes Dev 14: 2976–2988. 11114887 10.1101/gad.188700 PMC317095

[38] Dupaigne P, Tonthat NK, Espeli O, Whitfill T, Boccard F, et al (2012) Molecular basis for a protein-mediated DNA-bridging mechanism that functions in condensation of the E. coli chromosome. Mol Cell 48: 560–571. 23084832 10.1016/j.molcel.2012.09.009 PMC7505563

[39] Demarre G, Chattoraj DK (2010) DNA adenine methylation is required to replicate both Vibrio cholerae chromosomes once per cell cycle. PLoS Genet 6: e1000939. 20463886 10.1371/journal.pgen.1000939 PMC2865523

[40] Sliusarenko O, Heinritz J, Emonet T, Jacobs-Wagner C (2011) High-throughput, subpixel precision analysis of bacterial morphogenesis and intracellular spatio-temporal dynamics. Mol Microbiol 80: 612–627. 21414037 10.1111/j.1365-2958.2011.07579.x PMC3090749

[41] Marvig RL, Blokesch M (2010) Natural transformation of Vibrio cholerae as a tool–optimizing the procedure. BMC Microbiol 10: 155. 20509862 10.1186/1471-2180-10-155 PMC2890613

[42] Rudolph CJ, Upton AL, Stockum A, Nieduszynski CA, Lloyd RG (2013) Avoiding chromosome pathology when replication forks collide. Nature 500: 608–611. 23892781 10.1038/nature12312 PMC3819906

