# Supplementary figures and images for: Differential Management of the Replication Terminus Regions of the Two Vibrio cholerae Chromosomes during Cell Division
Source: PLoS Genet. 2014 Sep 25;10(9):e1004557. doi: 10.1371/journal.pgen.1004557 (PMC4177673; doi:10.1371/journal.pgen.1004557)

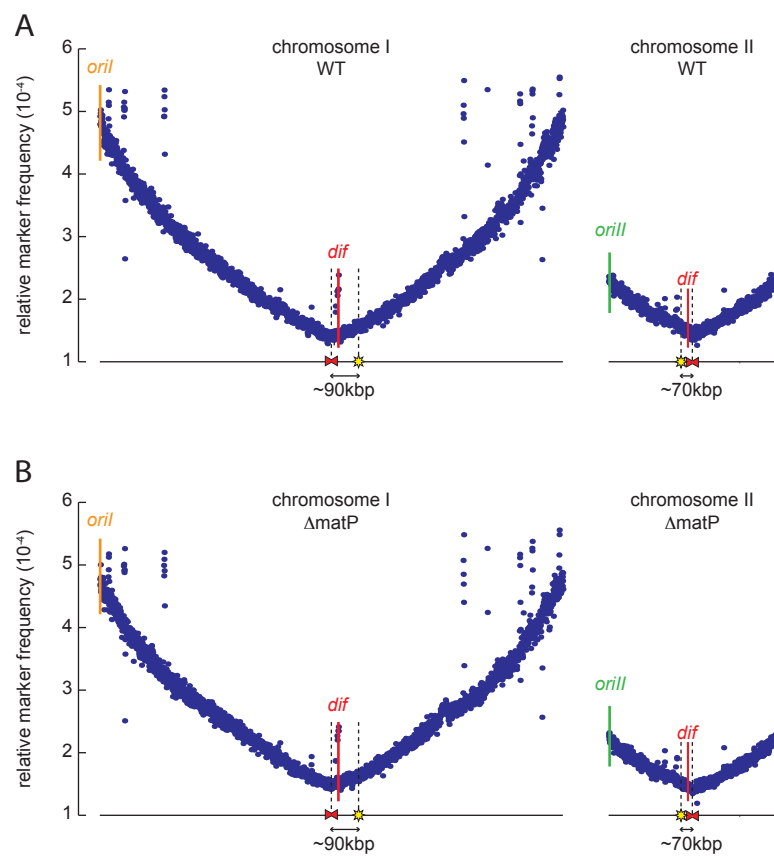

Supplementary Figure 1

Supplement: Figure S1 — Replication profiling of the two V. cholerae chromosomes in WT (A) and matP− (B) cells. To compare the timing of replication of chrI and chrII in the two genetic backgrounds, we calculated the relative frequency of uniquely mapping sequence tags within the genomic DNA of exponentially growing (replicating) cells in 1000 bp windows, i.e. the number of uniquely mapping sequence tags within each 1000 bp window divided by the total number of sequence that were uniquely mapped in the entire genome [42]. Left panels: chrI replication profiling; Right panels: chrII replication profiling. The position of the terminus is indicated by two facing red triangles. A yellow sun indicates the location of the single locus that had been so far visualized in the Ter of each of the two chromosomes. (PDF) [file pgen.1004557.s001.pdf]

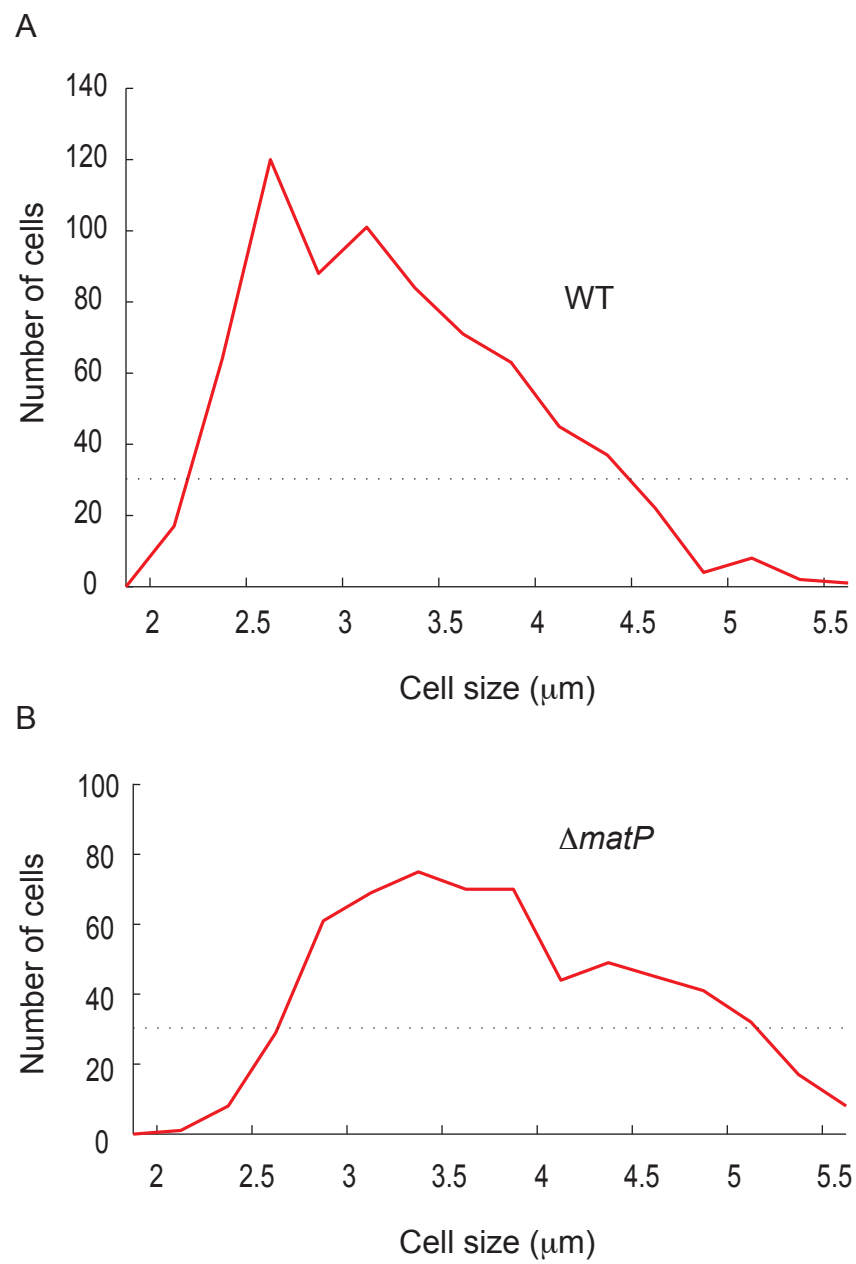

Supplementary Figure 2

Supplement: Figure S2 — Cell distribution in the WT strain (A) of V. cholerae or its matP− mutant (B). Cells were classified according to their length in bins of 0.25 µm. The dashed line shows the limit of 30 cells under which data was plotted in grey elsewhere in the manuscript. (PDF) [file pgen.1004557.s002.pdf]

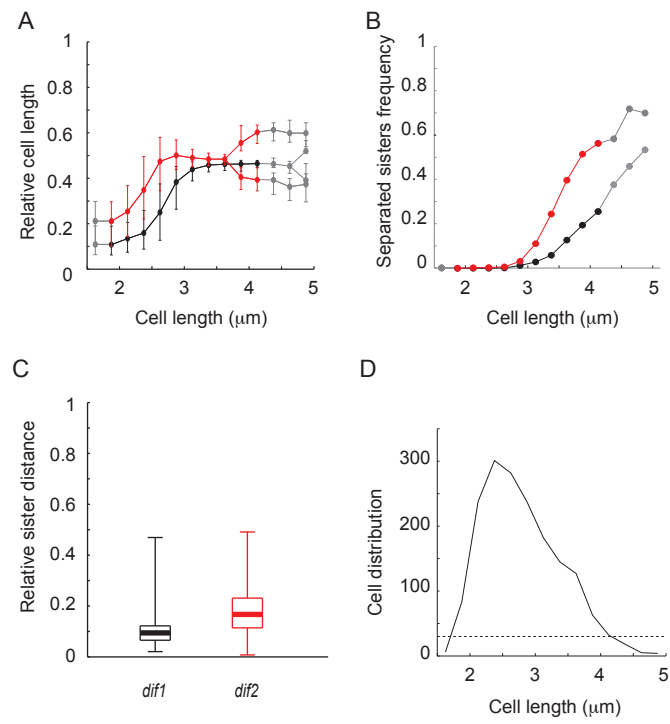

Supplementary Figure 3

Supplement: Figure S3 — Differences in the pattern of segregation of Ter I and Ter II are not due to the fluorescent microscopy visualization tools. The fluorescent markers that were used in Fig. 1 to label the dif1 and dif2 loci were switched: the dif1 locus was visualized using the YGFP–ParBPMT1/parS system and the dif2 locus was visualized with the lacO/LacI system. A. Relative position of dif1 (in black) and dif2 (in red) along the long axis of the cell as a function of cell length. B. Frequency of cells with separated dif1 (in black) and dif2 (in red) sisters as a function of cell length. The plain red and black lines show the data for the bins containing at least 30 cells; the dashed grey lines show the data for bins containing 3 to 29 cells. C. Interfocal distance of the sister copies of the dif locus of each of the two V. cholerae chromosomes, (dif1 in black and dif2 in red). D. Cell distribution. Cells were classified according to their length in bins of 0.25 µm. The dashed line shows the limit of 30 cells under which data was plotted. (PDF) [file pgen.1004557.s003.pdf]

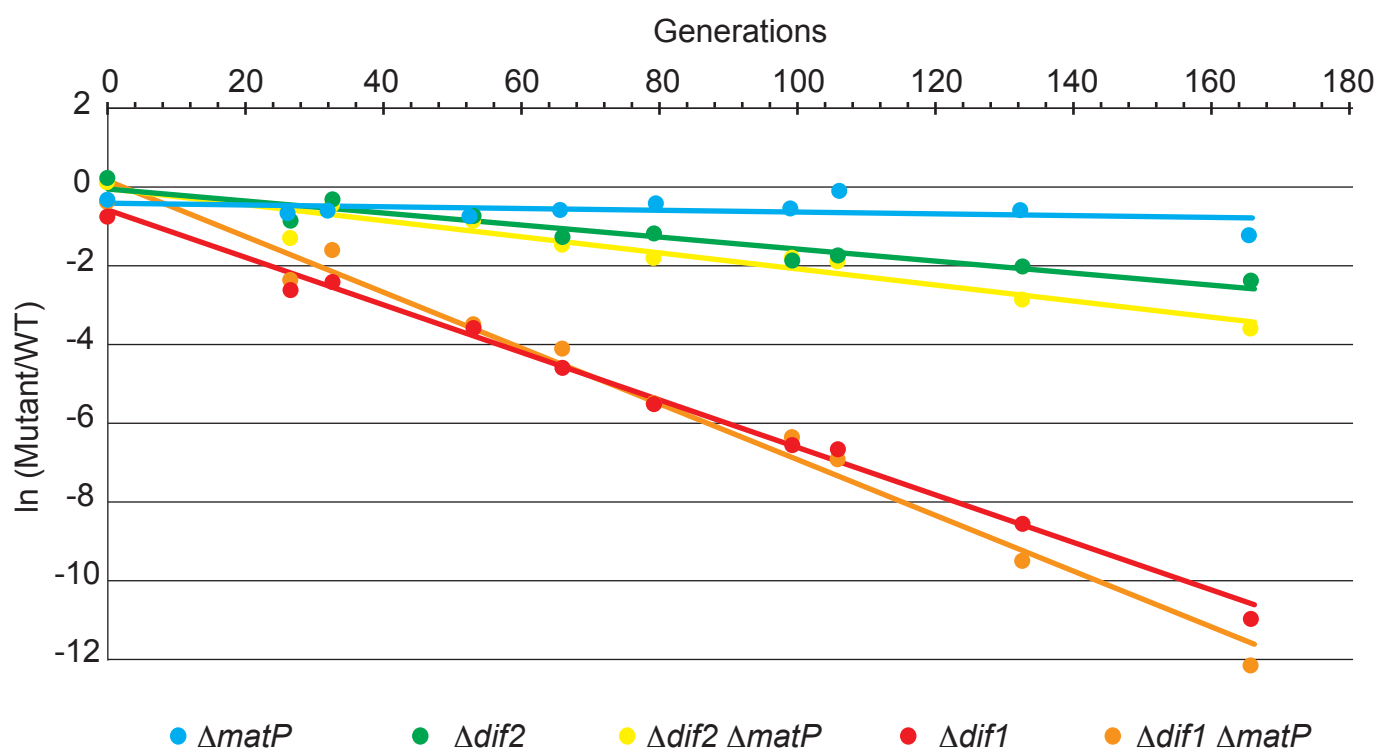

Supplementary figure 4

Supplement: Figure S4 — Graphic representation of growth competition between mutant strains of V. cholerae and a WT strain. The ratio of the mutant against its parental strain is plotted as a function of the number of generation. Cells were grown in parallel at 30°C with a 10−4 or a 10−5 dilution every 12 h for 5 days. Cell dilutions were plated every 24 h on cognate antibiotic plates to determine the number of CFU of the mutant versus the WT strain. The generation time between every time point was calculated from these numbers. The CFU ratio between mutant and its parental strain varies with the number of generations and it can be used to determine the loss of fitness of every mutant. The fitness loss for matP− cells was ≅0.23% (blue), for Δdif1 cells it was ≅6.9% (red), for Δdif1 matP− it was ≅5.9% (orange), for Δdif2 it was ≅2% (green) and for Δdif2 matP− it was ≅1.5 (yellow). (PDF) [file pgen.1004557.s004.pdf]

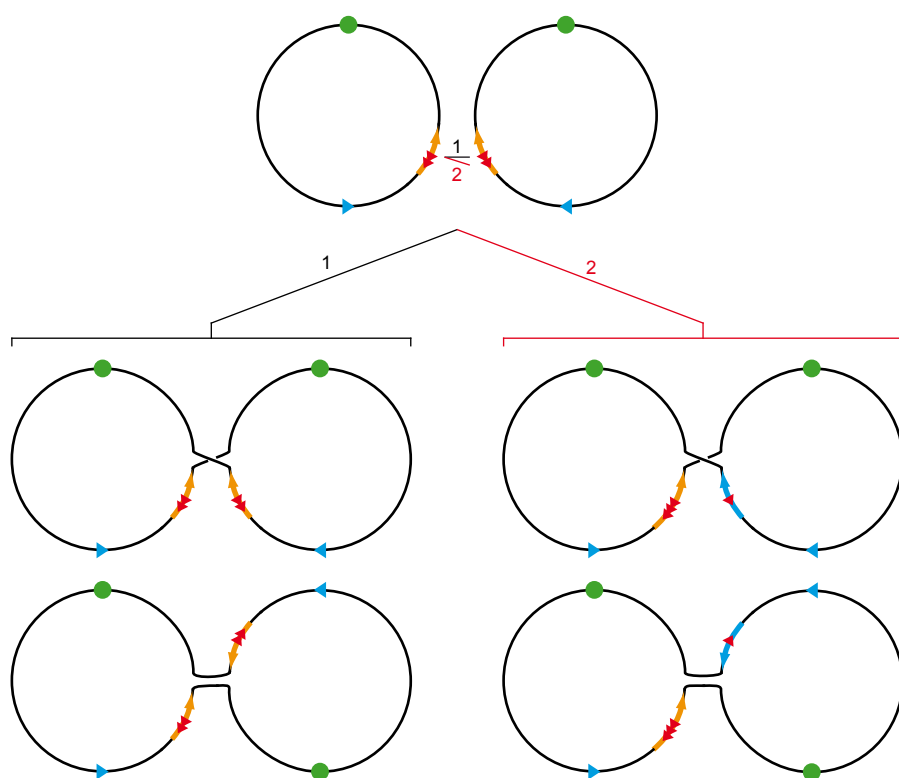

Supplementary Figure 5

Supplement: Figure S5 — Schematic representation of the possible intermolecular recombination events between lac2dif1 cassettes harboured on TerII sister chromatids. Green dot: oriII. Blue triangle: dif2. Red triangle: dif1. The orange arrow represents the lacZ gene disrupted by the two dif1 sites (lac2dif1) or three dif1 sites (lac3dif1). The blue arrows show the functional lacZ gene after the deletion of one dif1 site (lac1dif1). (PDF) [file pgen.1004557.s005.pdf]

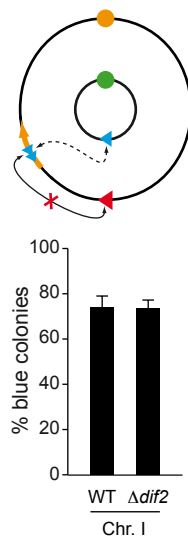

Supplementary Figure 6

Supplement: Figure S6 — Recombination between dif sites harboured on different chromosomes does not perturb the SCC detection. Schematic representation of the genome of a strain harbouring lac2dif2 on chI. No intrachromosomal recombination can occur between lac2dif2 and dif1 because of sequence incompatibility. The influence of chII dif2 on chI lac2dif2 recombination was tested by comparing results obtained in a strain in which dif2 was deleted. Results from at least three independent experiments. oriI represented with an orange dot and oriII by a green dot. dif1 is represented by a red triangle and dif2 with a blue triangle, the orange arrow show the lacZ gene disrupted by the two dif1 sites. (PDF) [file pgen.1004557.s006.pdf]

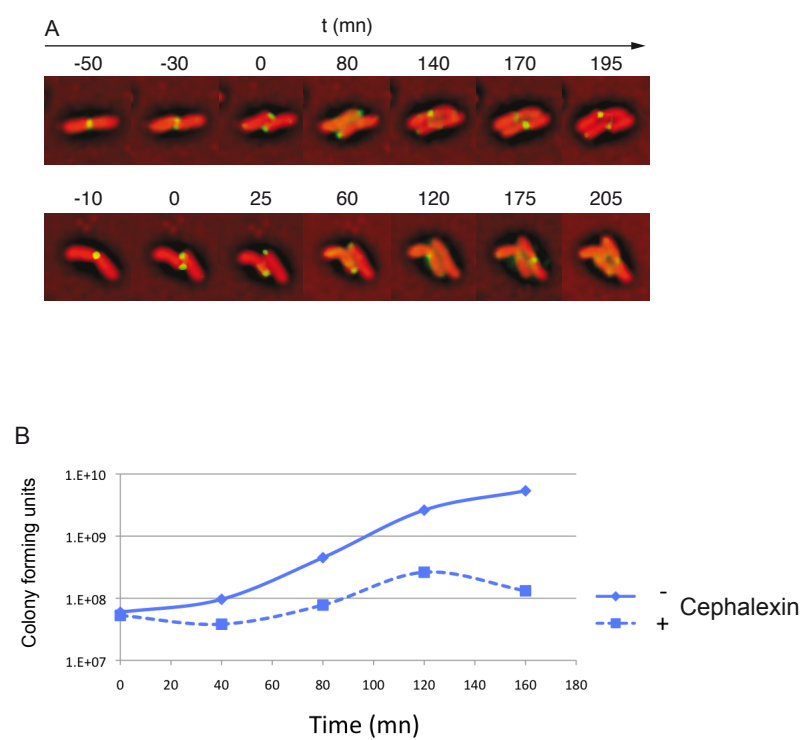

Supplementary Figure 7

Supplement: Figure S7 — (A) FtsK targets to midcell prior to cell division. Localization of FtsK-YFP in cells seen by video microscopy. The time before or after the first cell division event is indicated in minutes. (B) 2 h cephalexin treatment does not affect V. cholerae survival. Cells were grown without (plain line) or with (dashed line) cephalexin and spread on LB agar plates for cfu determination every 40 min. When cells were treated with cephalexin, the number of cfu didn't increase (as expected since cells can't divide) but remained constant. (PDF) [file pgen.1004557.s007.pdf]

A *dif2* locus

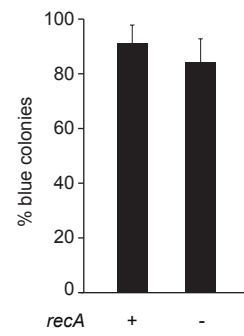

B  $\Delta matP$   
*dif* locus

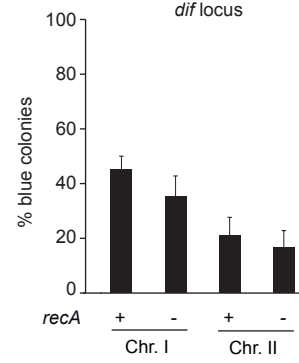

Supplementary Figure 8

Supplement: Figure S8 — Dimer formation does not influence SCC in V. cholerae. (A) Dimer formation does not influence SCC at dif2 in a WT background. The same is true at dif1 (Figure 3D). (B) Dimer formation does not influence SCC at dif1 and dif2 in a matP− background. Results from at least three independent experiments. (PDF) [file pgen.1004557.s008.pdf]

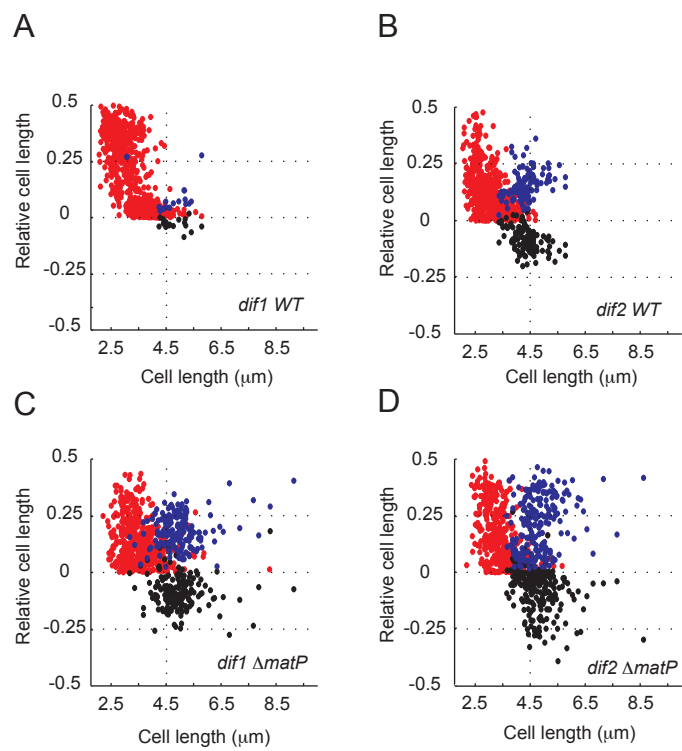

Supplementary Figure 9

Supplement: Figure S9 — Localization of dif1 and dif2 in WT and matP− strains of V. cholerae . dif1 (A and C) and dif2 (B and D) were localized in exponentially growing WT cells (GDV552; A and B ) and matP− cells (GDV564, C and D). The localization was done using the lacO/lacI-mcherry system at dif1 and YGFP–ParBPMT1/parS system at dif2. Plots show focus relative positions in cells with one (red) focus, and two (blue and black) foci as a function of the cell length. (PDF) [file pgen.1004557.s009.pdf]
