# Supplementary material for: Differential Management of the Replication Terminus Regions of the Two Vibrio cholerae Chromosomes during Cell Division
Source: PLoS Genet. 2014 Sep 25;10(9):e1004557. doi: 10.1371/journal.pgen.1004557 (PMC4177673; doi:10.1371/journal.pgen.1004557)
Supplement: Table S1 — List of bacterial strains. (DOCX) [file pgen.1004557.s012.docx]

**Supplementary Table 1**. List of bacterial strains

| **Name** | **Relevant genotype or features** | **Cassette** | | | **Reference** |
| --- | --- | --- | --- | --- | --- |
|  |  | **Type** | **Size** | **Position**  **Ch ; kb** |  |
| N16961 ChapR | N16961::mTn*7hapR* strep^R^ gm^R^ | none |  |  | [3] |
| ADV78 | N16961 *ChapR* Δ*lacZ ::(lacI-mcherry-YGFP-parBT1)*  *lacO-aph* inserted next to *dif1*  on chrI 53 | none |  |  | This study |
| EGV88 | EPV50 *matP*::*bla* amp^R^ | none |  |  | This study |
| EGV114 | N16961 Δ*lacZ ::(lacI-mcherry-YGFP-parBT1)*  *parST1-cat* inserted next to *dif1* cm^R^ | none |  |  | This study |
| EPV50 | N16961 *ChapR* Δ*lacZ* gm^R^ | none |  |  | This study |
| EPV271 | EPV50 *Δdif2*::*aadA1* spec^R^ | none |  |  | This study |
| EPV272 | EPV272 *matP*::*bla* spec^R^ amp^R^ | none |  |  | This study |
| EPV274 | EPV50 *Δdif1*::*aadA1* spec^R^ | none |  |  | This study |
| EPV276 | EPV272 *matP*::*bla* spec^R^ amp^R^ | none |  |  | This study |
| FXV1a | GDV21 *ftsK-GFP- Sh ble* zeo^R^ | none |  |  | This study |
| GDV21 | N16961 *ChapR* Δ*lacZ* strep^R^ gm^R^ | none |  |  | This study |
| GDV28 | GDV21 *xerC_ind_-aadA1* spec^R^ | none |  |  | This study |
| GDV192 | GDV28 cm^R^ | dif2-dif2 | 55bp | I ; 1564 (*dif1*) | This study |
| GDV210 | GDV28 cm^R^ | dif2-dif2 | 1kb | I ; 1564 (*dif1*) | This study |
| GDV211 | GDV28 cm^R^ | dif2-dif2 | 55pb | I ; 1564 (*dif1*) | This study |
| GDV221 | GDV21 *ΔxerC ::arr2* rif^R^ | none |  |  | This study |
| GDV235 | GDV28 cm^R^ | dif1-dif1 | 55bp | II ; 507 (*dif2*) | This study |
| GDV236 | GDV28 cm^R^ | dif1-dif1 | 1kb | II ; 507 (*dif2*) | This study |
| GDV237 | GDV28 *Δdif2* cm^R^ | dif1-dif1 | 55bp | II ; 507 (*dif2*) | This study |
| GDV238 | GDV28 *Δdif2* cm^R^ | dif1-dif1 | 1kb | II ; 507 (*dif2*) | This study |
| GDV241 | GDV28 *ΔrecA ::aph* km^R^ cm^R^ | dif1-dif1 | 55bp | II ; 507 (*dif2*) | This study |
| GDV242 | GDV28 *ΔrecA ::aph* km^R^ cm^R^ | dif1-dif1 | 1kb | II ; 507 (*dif2*) | This study |
| GDV243 | GDV28 *Δdif2 ΔrecA ::aph* km^R^ cm^R^ | dif1-dif1 | 55bp | II ; 507 (*dif2*) | This study |
| GDV244 | GDV28 *Δdif2 ΔrecA ::aph* km^R^ cm^R^ | dif1-dif1 | 1kb | II ; 507 (*dif2*) | This study |
| GDV245 | GDV28 *Ftsk_ΔC_ ::arr2* cm^R^ rif^R^ | dif1-dif1 | 55bp | II ; 507 (*dif2*) | This study |
| GDV246 | GDV28 *Ftsk_ΔC_ ::arr2* cm^R^ rif^R^ | dif1-dif1 | 1kb | II ; 507 (*dif2*) | This study |
| GDV247 | GDV28 *Δdif2 Ftsk_ΔC_ ::arr2* cm^R^ rif^R^ | dif1-dif1 | 55bp | II ; 507 (*dif2*) | This study |
| GDV252 | GDV28 *Δdif1* cm^R^ | dif2-dif2 | 1kb | I ; 1564 (*dif1*) | This study |
| GDV257 | GDV28 *Δdif1 ΔrecA ::aph* km^R^ cm^R^ | dif2-dif2 | 1kb | I ; 1564 (*dif1*) | This study |
| GDV278 | GDV28 *Δdif2* cm^R^ | dif1-dif1 | 55bp | II ; 507 (*dif2*) | This study |
| GDV279 | GDV28 *Δdif2* cm^R^ | dif2-dif2 | 55bp | II ; 507 (*dif2*) | This study |
| GDV306 | GDV28 cm^R^ | dif2-dif2 | 55bp | I ; 1551 | This study |
| GDV308 | GDV28 cm^R^ | dif2-dif2 | 55bp | I ; 1543 | This study |
| GDV310 | GDV28 cm^R^ | dif2-dif2 | 55bp | I ; 1519 | This study |
| GDV312 | GDV28 cm^R^ | dif2-dif2 | 55bp | I ; 1462 | This study |
| GDV314 | GDV28 cm^R^ | dif1-dif1 | 55bp | II ; 591 | This study |
| GDV316 | GDV28 cm^R^ | dif1-dif1 | 55bp | II ; 498 | This study |
| GDV330 | GDV28 *Δdif2 matP ::sh ble* zeo^R^ cm^R^ | dif1-dif1 | 55bp | II ; 507 (*dif2*) |  |
| GDV331 | GDV28 *Δdif1* cm^R^ | dif2-dif2 | 55bp | I ; 1564 (*dif1*) | This study |
| GDV334 | GDV28 *Δdif1 ΔrecA ::aph* km^R^ cm^R^ | dif2-dif2 | 55bp | I ; 1564 (*dif1*) | This study |
| GDV436 | GDV28 *Δdif1 Δdif2* cm^R^ | dif1-dif1 | 55bp | II ; 507 (*dif2*) | This study |
| GDV450 | GDV28 cm^R^ | dif1-dif1 | 55bp | II ; 659 | This study |
| GDV458 | GDV28 cm^R^ | dif1-dif1 | 55bp | II ; 1049 | This study |
| GDV460 | GDV28 cm^R^ | dif1-dif1 | 55bp | II ; 821 | This study |
| GDV471 | GDV28 cm^R^ | dif1-dif1 | 55bp | II ; 312 | This study |
| GDV486 | GDV28 *Δdif2* *Ftsk_ΔC_ ::arr2* rif^R^ cm^R^ | dif1-dif1 | 55bp | II ; 507 (*dif2*) | This study |
| GDV489 | GDV28 *Δdif2* *Ftsk_ΔC_ ::arr2* rif^R^ cm^R^ | dif2-dif2 | 55bp | II ; 507 (*dif2*) | This study |
| GDV530 | GDV28 *matP ::sh ble* zeo^R^ cm^R^ | dif1-dif1 | 55bp | II ; 591 | This study |
| GDV532 | GDV28 *matP ::sh ble* zeo^R^ cm^R^ | dif1-dif1 | 55bp | II ; 498 | This study |
| GDV534 | GDV28 *matP ::sh ble* zeo^R^ cm^R^ | dif1-dif1 | 55bp | II ; 659 | This study |
| GDV536 | GDV28 *matP ::sh ble* zeo^R^ cm^R^ | dif1-dif1 | 55bp | II ; 312 | This study |
| GDV552 | N16961 Δ*lacZ ::(lacI-mcherry-YGFP-parBT1)*  *lacO-aph* next to *dif1*  *parST1-cat* inserted next to *dif2* kn^R^ cm^R^ | none |  |  | This study |
| GDV564 | GDV552 *matP::sh ble* zeo^R^ kn^R^ cm^R^ | none |  |  | This study |
| GDV593 | GDV28 cm^R^ | dif2-dif2 | 55bp | I ; 1898 | This study |
| GDV595 | GDV28 cm^R^ | dif2-dif2 | 55bp | I ; 1099 | This study |
| GDV636 | GDV28 *matP::sh ble* zeo^R^ cm^R^ | dif1-dif1 | 55bp | II ; 821 | This study |
| GDV642 | GDV28 cm^R^ | dif2-dif2 | 55bp | I ; 1686 | This study |
| GDV666 | GDV28 *Δdif2* *Ftsk_KOPSblind_ -arr2* rif^R^ cm^R^ | dif1-dif1 | 55bp | II ; 507 (*dif2*) | This study |
| GDV668 | GDV28 *Δdif2* *Ftsk_KOPSblind_ ::arr2* rif^R^ cm^R^ | dif1-dif1 | 55bp | II ; 507 (*dif2*) | This study |
| GDV670 | GDV28 *Δdif1 Ftsk_KOPSblind_ ::arr2* rif^R^ cm^R^ | dif2-dif2 | 55bp | I ; 1564 (*dif1*) | This study |
