# Supplementary material for: Differential Management of the Replication Terminus Regions of the Two Vibrio cholerae Chromosomes during Cell Division
Source: PLoS Genet. 2014 Sep 25;10(9):e1004557. doi: 10.1371/journal.pgen.1004557 (PMC4177673; doi:10.1371/journal.pgen.1004557)
Supplement: Table S2 — List of plasmids. (DOCX) [file pgen.1004557.s013.docx]

**Supplementary Table 2.** List of plasmids

| **Name** | **Relevant genotype or features** | **Reference** |
| --- | --- | --- |
| pAD21 | *tet- Sh ble -tet* flanked by 2 homologous regions surrounding coordinate II,312kb, oriR6K, cm^R^ zeo^R^ | This study |
| pAD23 | *dif1-tet- Sh ble -tet* flanked by 2 homologous regions surrounding *dif1*, oriV, amp^R^ zeo^R^ | This study |
| pAD24 | *tet- Sh ble -tet* flanked by 2 homologous regions surrounding dif1, oriV, amp^R^ zeo^R^ | This study |
| pAD30a | *dif2- tet- Sh ble -tet* flanked by 2 homologous regions surrounding d*if2*, oriV, amp^R^ zeo^R^ | This study |
| pAD30b | *dif2- tet- Sh ble-tet* flanked by 2 homologous regions surrounding *dif2*, oriV, amp^R^ zeo^R^ | This study |
| pAD31a | *tet- Sh ble -tet* flanked by 2 homologous regions surrounding *dif2*, oriV, amp^R^ zeo^R^ | This study |
| pAD31b | *tet- Sh ble -tet* flanked by 2 homologous regions surrounding *dif2*, oriV, amp^R^ zeo^R^ | This study |
| pGD135 | Transposon, oriR6K, km^R^, oriTRP4 | This study |
| pGD162 | *Sh ble* flanked by *tet*, oriR6K, cm^R^ zeo^R^ | This study |
| pGD165 | 1kb *dif2-dif2* cassette flanked by tet, oriR6K, cm^R^ | This study |
| pGD200 | pUC18 :: *Δdif1::arr2*, oriV, rif^R^ amp^R^ | This study |
| pGD203 | 27bp *dif1-dif1* cassette flanked by tet, oriR6K, cm^R^ | This study |
| pGD208 | 27bp *dif2-dif2* cassette flanked by tet, oriR6K, cm^R^ | This study |
| pGD233 | pSC101 :: *ftsk*_KOPSBLIND_-*arr2,* rep, rif^R^ amp^R^ | This study |
| pGD243 | pDS132 :: *matP::bla* oriR6K, cm^R^, amp^R^ | This study |
| pJB31 | pDS132 :: *ΔxerC::araC-xerC-lacI-aadA1*, oriR6K, spec^R^, cm^R^ | This study |
| pMEV69 | pDS132 :: *ΔlacZ,* oriR6K, cm^R^ | This study |
| pMEV71 | pDS132 :: *Δdif2::aadA1* *,* oriR6K, spec^R^ cm^R^ |  |
|  |  |  |
| pMEV72 | pDS132 :: *ΔxerC::aadA1* *,* oriR6K, spec^R^ cm^R^ | This study |
| pMEV96 | pUC18 :: *ΔrecA::aph,* oriV, km^R^ amp^R^ | This study |
| pMEV131 | pDS132 :: *Δdif1::aadA1* *,* oriR6K, spec^R^ cm^R^ |  |
| pMEV232 | pUC18 :: *ftsk*_ΔC_*::arr2,* oriV, rif^R^ amp^R^ | This study |
| pMEV235 | pDS132 :: *Δdif2::arr2* ,oriR6K, rif^R^ cm^R^ | This study |
| pMEV245 | pDS132 :: *ΔxerC::arr2*; oriR6K, rif^R^ cm^R^ | This study |
| pSH4A | pUC18 :: *tet- Sh ble -tet* flanked by 2 homologous regions surrounding coordinate I,1551kb, oriV, amp^R^ zeo^R^ | This study |
| pSH5B | pUC18 :: *tet- Sh ble -tet* flanked by 2 homologous regions surrounding coordinate I,1543kb, oriV, amp^R^ zeo^R^ | This study |
| pSH6B | pUC18 :: *tet- Sh ble -tet* flanked by 2 homologous regions surrounding coordinate I,1519kb, oriV, amp^R^ zeo^R^ | This study |
