# Supplementary material for: Differential Management of the Replication Terminus Regions of the Two Vibrio cholerae Chromosomes during Cell Division
Source: PLoS Genet. 2014 Sep 25;10(9):e1004557. doi: 10.1371/journal.pgen.1004557 (PMC4177673; doi:10.1371/journal.pgen.1004557)
Supplement: Text S1 — Genetic engineering and microscopic analysis methods. (DOCX) [file pgen.1004557.s014.docx]

**Text S1**

***V. cholerae* genetic engineering**

All *V. cholerae* mutants were constructed by integration/excision or natural transformation. To this end, a derivative of the El Tor *V. cholerae* N16961 was rendered competent by the insertion of *hapR* by specific transposition [1]. Engineered strains were confirmed by PCR and by sequencing of the junctions.

**Construction of an insertion library and insertion of the recombination cassette around chromosome I and II**

A library of the N16961 *V. cholerae* strain was built using a mariner transposon to integrate the *tetR* gene using tetracyclin resistance as a selection. The insertion was mapped in 100 of the clones by direct sequencing of the DNA flanking the point of insertion by arbitrary-random PCR [2]. Out of this collection, we chose clones in which the *tetR* insertions occurred outside an open reading frame and outside an obvious promoter. These insertions were checked by PCR and their unicity by southern blot. While *tetR* confered sufficient resistance for the construction of the library, it failed as a selective marker for natural transformation. Therefore, we inserted the *zeo* gene within the *tetR* sequence of each of the clones of interest of our library using pGD162. Resistance to zeocin could then be used to recover the positions into GDV28 by natural transformation. The *tet-zeo-tet* cassette was introduced at the following coordinates of chromosome I, 53.36 kbp, 427 kbp, 613 kbp, 798 kbp, 1100 kbp, 1462 kbp, 1686 kbp, 1898 kbp, 2088 kbp, 2237 kbp and 2502 kbp; and at the following coordinates of chromosome II 498 kbp, 591 kbp, 659 kbp, 821 kbp and 1049 kb. These positions were completed by the insertion of the *tet-zeo-tet* cassette at 1519 kbp, 1543 kbp and 1551 kbp on chromosome I and at 312 kbp on chromosome II using vectors carrying the homology regions surrounding the relevant coordinates. Finally, specific vectors were constructed to insert the *tet-zeo-tet* cassette at the *dif1* locus and at the *dif2* locus, with or without deleting the original *dif* site and, in the case of the *dif*2 locus, into the two possible orientations.

|  |  |  |  |  |
| --- | --- | --- | --- | --- |

**Reference**

1. Marvig RL, Blokesch M (2010) Natural transformation of Vibrio cholerae as a tool--optimizing the procedure. BMC Microbiol 10: 155.

2. O'Toole GA, Kolter R (1998) Initiation of biofilm formation in Pseudomonas fluorescens WCS365 proceeds via multiple, convergent signalling pathways: a genetic analysis. Mol Microbiol 28: 449-461.

3. Meibom KL, Blokesch M, Dolganov NA, Wu CY, Schoolnik GK (2005) Chitin induces natural competence in Vibrio cholerae. Science 310: 1824-1827.

4. Rudolph CJ, Upton AL, Stockum A, Nieduszynski CA, Lloyd RG (2013) Avoiding chromosome pathology when replication forks collide. Nature 500: 608-611.
